# Gene Network Dysregulation in the Trigeminal Ganglia and Nucleus Accumbens of a Model of Chronic Migraine-Associated Hyperalgesia

**DOI:** 10.3389/fnsys.2018.00063

**Published:** 2018-12-18

**Authors:** Hyeonsoo Jeong, Laura S. Moye, Bruce R. Southey, Alvaro G. Hernandez, Isaac Dripps, Elena V. Romanova, Stanislav S. Rubakhin, Jonathan V. Sweedler, Amynah A. Pradhan, Sandra L. Rodriguez-Zas

**Affiliations:** ^1^Department of Animal Sciences, University of Illinois at Urbana-Champaign, Urbana, IL, United States; ^2^Department of Psychiatry, University of Illinois at Chicago, Chicago, IL, United States; ^3^Roy J. Carver Biotechnology Center, University of Illinois at Urbana-Champaign, Urbana, IL, United States; ^4^Department of Chemistry and the Beckman Institute, University of Illinois at Urbana-Champaign, Urbana, IL, United States; ^5^Department of Statistics, University of Illinois at Urbana-Champaign, Urbana, IL, United States

**Keywords:** hyperalgesia, migraine, RNA-seq, transcription factor, immune response, synapse processes, trigeminal ganglia

## Abstract

The pharmacological agent nitroglycerin (NTG) elicits hyperalgesia and allodynia in mice. This model has been used to study the neurological disorder of trigeminovascular pain or migraine, a debilitating form of hyperalgesia. The present study validates hyperalgesia in an established mouse model of chronic migraine triggered by NTG and advances the understanding of the associated molecular mechanisms. The RNA-seq profiles of two nervous system regions associated with pain, the trigeminal ganglia (TG) and the nucleus accumbens (NAc), were compared in mice receiving chronic NTG treatment relative to control (CON) mice. Among the 109 genes that exhibited an NTG treatment-by-region interaction, solute carrier family 32 (GABA vesicular transporter) member 1 (*Slc32a1*) and preproenkephalin *(Penk*) exhibited reversal of expression patterns between the NTG and CON groups. Erb-b2 receptor tyrosine kinase 4 (*Erbb4*) and solute carrier family 1 (glial high affinity glutamate transporter) member 2 (*Slc1a2*) exhibited consistent differential expression between treatments across regions albeit at different magnitude. Period circadian clock 1 (*Per1*) was among the 165 genes that exhibited significant NTG treatment effect. Biological processes disrupted by NTG in a region-specific manner included adaptive and innate immune responses; whereas glutamatergic and dopaminergic synapses and rhythmic process were disrupted in both regions. Regulatory network reconstruction highlighted the widespread role of several transcription factors (including *Snrnp70, Smad1, Pax6, Cebpa*, and *Smpx*) among the NTG-disrupted target genes. These results advance the understanding of the molecular mechanisms of hyperalgesia that can be applied to therapies to ameliorate chronic pain and migraine.

## Introduction

Migraine affects approximately 15% of the world's population and the World Health Organization considers migraine a top ten most disabling conditions with migraineurs experiencing enhanced intensity of pain or hyperalgesia. The therapeutic options for chronic migraine sufferers are limited and provide incomplete symptom relief (Bigal et al., 2008). The nitric oxide (NO) donor nitroglycerin (NTG) has been used extensively to understand the nociceptive system and pain processing (Cury et al., 2011). Nitroglycerin provokes pain in migraine-susceptible patients (Christiansen et al., 1999; Afridi et al., 2005; Olesen, 2008), and has been shown to evoke hyperalgesia in rodents (Di et al., 2016; Ferrari et al., 2016; Tipton et al., 2016; Demartini et al., 2017).

The trigeminal ganglia (TG) and nucleus accumbens (NAc) are two nervous system regions associated with chronic migraine and pain perception (Akerman et al., 2011; Schwartz et al., 2014). In response to NO, TG neurons trigger vasodilation and neurogenic inflammation, which elicit hyperalgesia, sensitization, and allodynia (Bellamy et al., 2006; Akerman et al., 2011). Chronic pain and migraine signal other nervous system regions including disruption of the nucleus accumbens (NAc) processes that play a role in migraine and hyperalgesia co-morbidities including depression, irritability, fatigue, sleepiness, and loss of appetite (Burstein and Jakubowski, 2005; De Felice et al., 2013; Yuan et al., 2013; Elman and Borsook, 2016). Alterations in neural circuits in these nervous system regions have also been shown to play a role in medication overuse headache (Calabresi and Cupini, 2005; Torta et al., 2016) that can subsequently result in chronic migraine symptoms.

Nitroglycerin has been used extensively to model migraine-associated symptoms in rodents (Bates et al., 2010; Markovics et al., 2012; Pradhan et al., 2014a,b). Chronic intermittent administration of NTG has been developed as a model of chronic migraine (Pradhan et al., 2014a; Moye and Pradhan, 2017) and many groups reported that chronic NTG treatment results in chronic hyperalgesia, photophobia, and increased expression of migraine-related peptides (Greco et al., 2011; Pradhan et al., 2014b; Farajdokht et al., 2017; Moye and Pradhan, 2017; Long et al., 2018) In addition, this model has been pharmacologically validated, and migraine therapies such as the abortive, sumatriptan; and the preventives, topiramate, and propranolol inhibit the effects of NTG in mice (Pradhan et al., 2014a; Tipton et al., 2016; Moye and Pradhan, 2017). Further, NTG has also been shown to produce migraine-related symptoms including, light-aversive behavior (Markovics et al., 2012; Sufka et al., 2016; Farajdokht et al., 2017), and increased meningeal blood flow in rodents (Greco et al., 2011; Markovics et al., 2012).

Studies of NTG-treated rodents can offer important insights into the mechanisms of hyperalgesia that can shed light into solutions for chronic pain such as migraine (Bates et al., 2010; Ferrari et al., 2015; Pedersen et al., 2016; Sufka et al., 2016). A study of the effect of one acute NTG infusion in the TG of male rats uncovered 15 differentially expressed genes relative to controls including glutamine synthetase (*Glul*), period circadian clock 1 (*Per1*), and genes that participate in immune responses including TAP binding protein (*Tapbp*), RT1 class Ia, locus A2 (*RT1-A2*) and RT1 class I, locus A3 (*RT1-A3*) (Pedersen et al., 2016). The molecular mechanisms disrupted by chronic NTG treatment evoking hyperalgesia in mice are incompletely understood. A comprehensive study of these pathways can offer insights into effective therapies to alleviate chronic pain and migraine (Bates et al., 2010; Ferrari et al., 2015; Pedersen et al., 2016; Sufka et al., 2016).

The objective of this study is to advance the understanding of the molecular disruptions that occur in a chronic hyperalgesic state associated with migraine. A transcriptomic analysis was undertaken to identify genes, biological processes and regulatory networks impacted by chronic NTG exposure in the TG and NAc of mice. The characterization of gene and pathway dysregulation can offer insights into the molecular mechanisms disrupted by NTG-evoked hyperalgesia in mice. These findings can shed light into solutions for chronic pain conditions such as migraine (Bates et al., 2010; Ferrari et al., 2015; Pedersen et al., 2016; Sufka et al., 2016).

## Materials and Methods

### Animals Experiments

Male C57BL6/J mice (Jackson Laboratories, Bar Harbor, ME) between 9 and 12 weeks old were studied. Mice were group housed in a 12-12 light-dark cycle, and food was available *ad libitum*. All experimental procedures were approved by the University of Illinois at Chicago Office of Animal Care and Institutional Biosafety Committee, in accordance with AALAC guidelines and the Animal Care Policies of the University of Illinois at Chicago, as well as with the European Union directive on the subject of animal rights. Mice were weighed daily during treatment, and no adverse effects of treatment were observed on body weight.

Chronic migraine-associated pain was modeled using intraperitoneal injections of NTG (10 mg/kg, IP) every second day for 9 days totaling 5 test days (Pradhan et al., 2014a; Tipton et al., 2016). The NTG injection was prepared daily from a stock solution of 5.0 mg/mL NTG and diluted in 0.9% saline. Control mice received intraperitoneal injections of 0.9% saline and were treated in parallel to NTG-treated mice. Mice were randomly allocated to either chronic NTG (NTG) or control (CON) groups. Male mice were studied to remove the effect of the estrus cycle stage on the molecular disruptions triggered by NTG.

The hind paw and cephalic mechanical threshold experiments used separate groups of mice from the same population. For all behavioral experiments, mice were counterbalanced into treatment groups following the first basal test for mechanical sensitivity on naïve mice. The experimenter was blinded to the drug condition being tested, basal responses are assessed before daily injection and injection volume was 10 ml/kg. No adverse effects of injection were observed in any of the experiments. All mice were tested in a separate behavior room with low-light (~35–50 lux) and low-noise conditions, between 09:00 and 16:00. For all behavioral tests, mice were habituated to the testing rack for 2 days prior to the first test day, and on each test day for 20 minutes prior to the first measurement. For cephalic testing, mice were tested in 118 ml paper cups, to which they had been previously habituated for 1 h over 2 days.

The plantar surface of the mouse hind paw and the periorbital region caudal to the eyes and near the midline were tested for the peripheral and cephalic mechanical threshold measurements. To assess mechanical sensitivity, the threshold for responses to punctate mechanical stimuli (mechanical allodynia) was tested according to the up-and-down method (Chaplan et al., 1994). The region of interest was stimulated with a series of eight von Frey hair filaments (bending force ranging from 0.00g to 2g). A response was defined as a lifting, shaking, or licking of the hind paw or head, depending on the region tested. The first filament tested was 0.4g. In the absence of a response, a heavier filament (up) was tried, and in the presence of a response, a lighter filament (down) was tested. This pattern was followed for a maximum of four filaments following the first response. The peripheral threshold was measured on 9 consecutive days and the cephalic threshold was measured on days 1, 5, and 9. The effect of treatment was evaluated using a two way ANOVA including the effects of treatment, day and interaction and testing included the *post hoc* Holm-Sidak adjustment.

### RNA Extraction and Sequencing

Mice were anesthetized with pentobarbital (somnosol), euthanized, and intracardially perfused with ice-cold saline approximately 24 h after the last injection of NTG or saline. Brains were extracted and the TG and NAc were rapidly dissected, snap-frozen, and stored at −80°C. Total RNA was obtained from each collected nervous system region of an individual mouse following manufacturer's instructions. Steps included tissue homogenization with TRIzol (Invitrogen, Carlsbad, CA) and ceramic beads (MO BIO, Carlsbad, CA), and RNA isolation using the RNA-kit (Omega Biotek, Norcross, GA).

All 20 RNA samples representing 2 treatments (NTG and CON) and 2 nervous system regions had RNA integrity number values above 7.5 and were individually analyzed. Paired-end reads 100 nt in length were sequenced using the HiSeq 4000 (Illumina, San Diego, CA) platform. The RNA-seq datasets for this study are available in the National Center for Biotechnology Information Gene Expression Omnibus (GEO) database (identifier GSE110194). The average Phred quality score of the reads was assessed using FastQC (Andrews, 2010). The nucleotide quality score was >30 across all read positions. The read sequences were deemed of high quality and were not trimmed.

### RNA Quantification and Differential Expression Analysis

The paired-end reads of each of the samples were individually mapped to the C57Bl/6J mouse genome [version GRCm38, downloaded on October 2016 from NCBI (Pruitt et al., 2007)] using Kallisto (Bray et al., 2016) with default settings. Gene expression transcript counts were imported into R (version 3.2) for analysis using tximport (release 3.5) (Soneson et al., 2015) and analyzed with edgeR using default settings (Robinson et al., 2010). Gene expression transcript counts were analyzed using a generalized linear model including the main factors of treatment group (NTG or CON) and nervous system region (TG or NAc) and the interaction between treatment and region. All genes with 5 or more reads per treatment-region group were analyzed to ensure gene expression in all groups tested for differential expression. The Benjamini-Hochberg false discovery rate (FDR) was used to adjust the differential expression *P*-value for multiple testing (Benjamini and Hochberg, 1995).

### Functional Enrichment and Network Inference

Enrichment of Gene Ontology (GO) biological processes (BPs) and molecular functions (MFs) and KEGG pathways among the differentially expressed genes were evaluated using complementary methods (Reiner et al., 2003; Caetano-Anollés et al., 2015, 2016; Nixon et al., 2015; Gonzalez-Pena et al., 2016a,b). Differentially expressed genes were analyzed with the Database for Annotation, Visualization and Integrated Discovery (DAVID; Version 6.8) using the Direct GO terms available in this repository (Huang et al., 2009). The *Mus musculus* genome was used as background for testing. Enrichment of each category was assessed using the Expression Analysis Systematic Explorer (EASE) score that was computed based on a one-tailed jackknifed Fisher hypergeometric exact test. The clustering of functional categories facilitated the interpretation of enriched terms. The statistical significance of each cluster of categories was assessed using an enrichment score computed as the geometric mean of the -log_10_ EASE scores of categories within each cluster (Serão et al., 2013; Caetano-Anollés et al., 2015, 2016; Gonzalez-Pena et al., 2016a).

Additional insights into the functional categories impacted by the treatment and nervous system region were gained using the Gene Set Enrichment Analysis (GSEA) approach implemented in the software package GSEA-P (version 2.0) (Subramanian et al., 2007). GSEA-P provides an enrichment score of functional categories in the *Mus musculus* Molecular Signature Database (MSigDB) that is calculated using maximum deviation of cumulative sum based on the gene-phenotype correlation (Subramanian et al., 2007).

Genes exhibiting a significant interaction between treatment and nervous system region, significant treatment main effect, or significant region main effect were queried as potential targets of enriched transcription factors. Significantly differentially expressed genes within each group were searched against the database of target genes ranked according to the associated transcription factor motifs using the iRegulon (Verfaillie et al., 2014) plugin in the Cytoscape environment (Shannon et al., 2003). A transcription factor normalized enrichment score was computed for each group where a normalized enrichment score > 3.0 corresponds to an approximate FDR between 3 and 9% (Verfaillie et al., 2014).

## Results

### Study of Mechanical Pain

**Figure 1:**
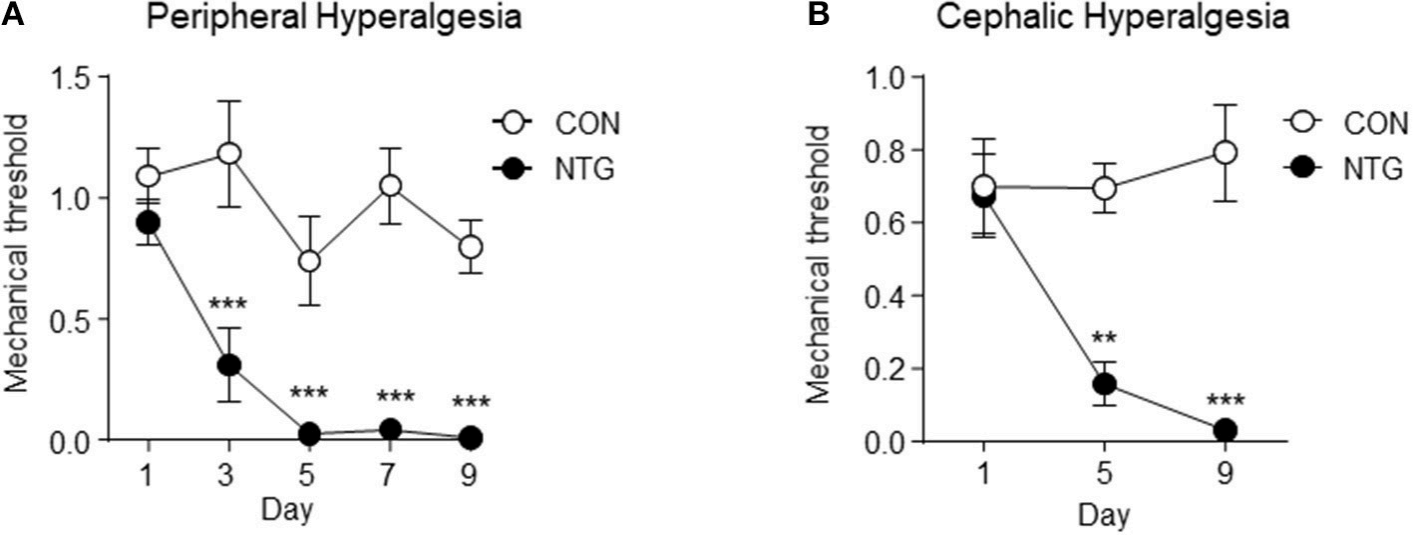
Peripheral **(A)** and cephalic **(B)** mechanical threshold indicator of hyperalgesia in the chronic nitroglycerin (NTG) or control (CON) mouse groups across day of treatment. Mechanical thresholds were determined before each injection using von Frey filaments. Two-way analysis of variance including the effects of treatment, day, and interaction; A. Hindpaw measurement (*n* = 5/group); treatment, day, interaction *P*-value < 0.01; ^***^ within day Holm-Sidak testing *P*-value < 0.001. **(B)** Periorbital region caudal to the eyes measurement (*n* = 6/group); treatment, day, interaction *P*-value < 0.05; ^**^ within day Holm-Sidak testing *P*-value < 0.01, ^***^P-value < 0.001. Results are represented as mean ± SEM.

Figure 1 depicts the profile of peripheral (left graph A) and cephalic (right graph B) mechanical threshold across the days when mice received via IP injection of NTG (black markers) or saline (white markers). The values depicted are the means and standard error of the mean (SEM) and the *P*-values include a Holm-Sidak adjustment for multiple comparison. The interaction between treatment and day, the main effect of treatment and the main effect of day were significant at *P*-value < 0.01. The contrasts between treatments within day were also significant at *P*-value < 0.001 (denoted with “^***^”) or *P*-value < 0.01 (denoted with “^**^”).

### Summary of RNA-Seq Measurements

Approximately 3 billion reads were generated across all 20 samples and approximately 70 million paired-end sequence readings were obtained per sample. No statistical difference in the number of mapped reads was detected between sample groups. The average percentage of the reads mapped to the mouse transcriptome was approximately 82% (±4%). The RNA-seq reads were mapped to transcripts and after filtering for low counts (<5 reads) 22,071 genes were analyzed for the effects of the interaction between NTG treatment and nervous system region, and the main effects of NTG treatment and region studied.

### Nitroglycerin Treatment-by-Nervous System Region Interaction Effect on Gene Expression

Twenty-five genes exhibited significant (FDR-adjusted *P*-value < 0.14 or *P*-value ≤ 2.2E-04) differential expression for the interaction between treatment and region effect (Table 1). An expanded list of 109 genes including the previous 25 genes and others exhibiting interaction effect is provided in Table S1. The majority of these genes (96 genes) were differentially expressed between the treatment groups in the TG whereas the remaining 13 genes were significantly differentially expressed between the treatments in the NAc (Table S1A).

**Table 1:** Gene profiles exhibiting interaction between nitroglycerin treatment and nervous system region effect on expression.

	**Overall**		**Comparisons between interaction treatment-region levels^b^**					
**Gene symbol**	***P*-value**	**FDR *P*-value^a^**	**NTG(NAc)- CON(NAc)**	**NTG(TG)- CON(TG)**	**CON(TG)- CON(NAc)**	**NTG(TG)- NTG(NAc)**	**NTG(TG)- CON(NAc)**	**CON(TG)- NTG(NAc)**
*Gm31690*	1.3E-08	3.0E-04	−0.09	2.31	−5.52	−3.09	−3.18	−5.43
*C030029H02Rik*	1.7E-07	1.9E-03	−0.17	2.00	−5.94	−3.80	−3.97	−5.77
*Lrrc8a*	6.5E-07	3.6E-03	0.11	−0.18	1.18	0.86	0.97	1.07
*Cdr2l*	3.5E-06	9.7E-03	0.15	−0.16	2.89	2.55	2.70	2.74
*Gm39080*	1.7E-05	4.1E-02	0.13	1.81	−4.44	−2.79	−2.65	−4.57
*AW047730*	1.9E-05	4.3E-02	0.06	1.77	−5.88	−4.19	−4.13	−5.93
*BC006965*	2.7E-05	5.5E-02	−0.17	1.68	−3.68	−1.86	−2.02	−3.51
*Opalin*	3.3E-05	5.5E-02	−0.20	2.02	−7.22	−5.02	−5.22	−7.01
*A230001M10Rik*	4.1E-05	6.5E-02	−0.14	1.09	−3.72	−2.50	−2.65	−3.57
*Scrg1*	5.7E-05	7.8E-02	0.06	1.44	−5.46	−4.09	−4.03	−5.52
*Gm39673*	7.0E-05	8.1E-02	−0.07	0.57	−0.67	−0.06	−0.12	−0.59
*Gm10052*	9.4E-05	9.9E-02	−0.13	1.05	−2.23	−1.07	−1.21	−2.09
*Gm29907*	1.0E-04	1.0E-01	0.53	−0.40	−0.24	−1.19	−0.66	−0.76
*Rpl23*	1.4E-04	1.2E-01	−0.23	0.23	−0.28	0.15	−0.07	−0.05
*Gm39728*	1.5E-04	1.2E-01	0.79	−0.82	−0.08	−1.71	−0.92	−0.86
*Ccdc190*	1.5E-04	1.2E-01	−0.23	0.73	−3.45	−2.51	−2.74	−3.21
*Neu4*	1.5E-04	1.2E-01	0.02	1.43	−4.97	−3.56	−3.54	−4.98
*Gm42386*	1.6E-04	1.2E-01	−0.12	0.95	−3.32	−2.27	−2.39	−3.19
*Camkv*	1.6E-04	1.2E-01	0.02	0.67	−7.75	−7.13	−7.11	−7.77
*H2afz*	1.7E-04	1.2E-01	−0.23	0.09	−0.48	−0.19	−0.41	−0.25
*Slc32a1*	2.1E-04	1.4E-01	−0.03	1.94	−9.26	−7.32	−7.35	−9.22
*Gm20594*	2.2E-04	1.4E-01	−0.08	0.31	−3.31	−2.95	−3.03	−3.23
*Efnb3*	2.2E-04	1.4E-01	−0.10	0.76	−4.20	−3.36	−3.46	−4.09

a *False discovery rate (FDR) adjusted P-value for the overall treatment by region interaction*.

b *Log_2_ (fold change) between different pairs of treatment and region groups: NTG(TG), trigeminal ganglia (TG) from nitroglycerin-treated (NTG) mice; NTG(NAc): nucleus accumbens (NAc) from NTG mice; CON(TG): TG from Control (CON) mice; and CON(NAc): NAc from CON mice*.

Three main patterns can be identified among the genes exhibiting an interaction effect (Table 1, Table S1). The first pattern has opposite differential expression between treatments across regions (87 genes). Among the genes over-expressed in the TG of NTG-treated relative to CON mice that exhibit a reverse profile in NAc were: oligodendrocytic myelin paranodal and inner loop protein (*Opalin*); ephrin B3 (*Efnb3*); solute carrier family 32 gamma amino butyrate (GABA vesicular transporter), member 1 (*Slc32a1*); preproenkephalin (*Penk*); and RAR-related orphan receptor beta (*Rorb*). Among the genes over-expressed in the NAc of NTG-treated compared to CON mice were: Leucine-rich repeat containing 8A (*Lrrc8a*); calcium channel, voltage-dependent, N type, alpha 1B subunit (*Cacna1b*); histocompatibility 2 (class II) antigen E beta2 (*H2-Eb2*); predicted gene 1673 neuropeptide-like protein C4orf48 homolog isoform 1 (*Gm1673*); and egl-9 family hypoxia-inducible factor 2 (*Egln2*).

The second pattern has consistent relative expression between treatments albeit the significance level differed between the investigated regions (20 genes). Among the genes exhibiting consistent relative expression between treatments across nervous system regions albeit at different magnitude levels were: POU domain, class 3, transcription factor 3 (*Pou3f3*); erb-b2 receptor tyrosine kinase 4 (*Erbb4*); and solute carrier family 1 (glial high affinity glutamate transporter), member 2 (*Slc1a2*).

The final pattern was characterized by significant differential expression in one region with no differential expression in another region (3 genes). Genes exhibiting this profile included: natriuretic peptide type C (*Nppc*) prohormone, and two long noncoding RNA genes (*Gm15738* and *Gm39717*).

### Functional Analysis of the Genes Exhibiting Nitroglycerin Treatment-by-Nervous System Region Interaction Effects

Enrichment analysis revealed two clusters of enriched GO categories associated with positive regulation of transcription (enrichment score = 1.8 and 1.5, respectively) that were identified among the genes exhibiting significant treatment-by-nervous system region interaction effects (Table 2). Table S2 includes these clusters and additional enriched GO categories and KEGG pathways among genes exhibiting treatment-by-region interaction effect.

**Table 2:** Clusters of informative Gene Ontology (GO) categories enriched (DAVID enrichment score ES ≥ 1.5) among genes exhibiting significant nitroglycerin treatment-by-nervous system regions interaction effects.

**GO category^a^**	**GO identifier**	**GO name**	***P*-value**
**Cluster 1 (ES: 1.8)**			
BP	GO:0045893	Positive regulation of transcription, DNA-templated	5.8E-03
MF	GO:0003682	Chromatin binding	2.7E-02
MF	GO:0044212	Transcription regulatory region DNA binding	3.4E-02
**Cluster 2 (ES:1.5)**			
BP	GO:0045893	Positive regulation of transcription, DNA-templated	5.8E-03
MF	GO:0003677	DNA binding	7.0E-03
MF	GO:0043565	Sequence-specific DNA binding	9.6E-03
BP	GO:0006351	Transcription, DNA-templated	2.2E-02
MF	GO:0003700	Transcription factor activity, sequence-specific DNA binding	7.8E-02

a *BP, biological process; MF, molecular function*.

Complementary GSEA used information on differential expression between NTG and CON treatments within the studied regions. Tables 3, 4 present the most enriched (*P*-value < 0.005; normalized enrichment score > |1.7|) categories, selected to minimize redundancies, in the TG and NAc respectively, and the corresponding Tables S3, S4 present extended and complete lists of categories.

**Table 3:** Gene Ontology (GO) categories enriched (GSEA normalized enrichment score NES > |1.72|) among genes exhibiting significant differential expression between nitroglycerin-treated and control mice in the trigeminal ganglia.

**GO category^a^**	**GO identifier**	**Name**	**NES^b^**	***P*-value**	**FDR *P*-value^c^**
**UNDER-EXPRESSED IN NITROGLYCERIN-TREATED RELATIVE TO CONTROL MICE**					
MF	GO:0000287	Magnesium ion binding	−1.85	0.0E+00	6.4E-02
MF	GO:0001882	Nucleoside binding	−1.72	0.0E+00	2.4E-01
**OVER-EXPRESSED IN NITROGLYCERIN-TREATED RELATIVE TO CONTROL MICE**					
BP	GO:0002460	Adaptive immune response	1.75	0.0E+00	1.0E-01
BP	GO:0001913	T cell mediated cytotoxicity	1.86	4.9E-03	8.3E-02
BP	GO:0002449	Lymphocyte mediated immunity	1.82	0.0E+00	9.0E-02
BP	GO:0019724	B cell mediated immunity	1.76	0.0E+00	9.7E-02
BP	GO:0007260	Tyrosine phosphorylation of STAT protein	1.81	6.9E-03	7.6E-02
BP	GO:0010906	Regulation of glucose metabolic process	1.85	0.0E+00	7.1E-02
BP	GO:0021983	Pituitary gland development	1.87	2.3E-03	1.2E-01
BP	GO:0007601	Visual perception	1.74	0.0E+00	1.0E-01
BP	GO:0050953	Sensory perception of light stimulus	1.71	0.0E+00	1.3E-01

a *BP, biological process; MF, molecular function*.

b *Normalized enrichment score where positive and negative value refer to over- and under-expressed in nitroglycerin-treated relative to control mice, respectively*.

c *False discovery rate (FDR) adjusted P-value*.

**Table 4:** Gene Ontology (GO) categories enriched (GSEA normalized enrichment score NES > |1.74|) among genes exhibiting significant differential expression between nitroglycerin-treated and control mice in the nucleus accumbens.

**GO category^a^**	**GO identifier**	**Name**	**NES^b^**	***P*-value**	**FDR *P*-value^c^**
**UNDER-EXPRESSED IN NITROGLYCERIN-TREATED RELATIVE TO CONTROL MICE**					
BP	GO:2000377	Regulation of reactive oxygen species metabolic process	−1.76	0.0E+00	2.1E-01
BP	GO:0034341	Response to interferon gamma	−1.76	2.2E-03	1.6E-01
BP	GO:0045087	Innate immune response	−1.74	0.0E+00	1.7E-01
**OVER-EXPRESSED IN NITROGLYCERIN-TREATED RELATIVE TO CONTROL MICE**					
BP	GO:0060079	Regulation of excitatory postsynaptic membrane potential	1.84	0.0E+00	1.0E-01
BP	GO:0051606	Detection of stimulus	1.81	0.0E+00	1.4E-01
MF	GO:0005230	Extracellular ligand gated ion channel activity	1.79	0.0E+00	1.2E-01
BP	GO:0007215	Glutamate receptor signaling pathway	1.78	0.0E+00	9.4E-02
BP	GO:0009581	Detection of external stimulus	1.76	0.0E+00	9.7E-02
BP	GO:0001964	Startle response	1.69	5.4E-03	1.6E-01
BP	GO:0050906	Detection of stimulus involved in sensory perception	1.69	5.5E-03	1.6E-01

a *BP, biological process; MF, molecular function*.

b *Normalized enrichment score where positive and negative value refer to over- and under-expressed in nitroglycerin-treated relative to control mice, respectively*.

c *False discovery rate (FDR) adjusted P-value*.

Within the TG (Table 3), enriched categories among the genes over-expressed in the NTG group include: adaptive immune response (GO:0001913; GO:0002449; GO0019724; GO:0002460); regulation of glucose metabolic process (GO:0010906); pituitary gland development (GO:0021983); visual and light perception (GO:00076601; GO:0050953); and retina processes (GO:0060041, GO:0060042). Functional categories enriched among the genes under-expressed in the TG of NTG-treated relative to CON mice (Table 4) were the innate immune response category (GO:0045087) and regulation of reactive oxygen species (GO:2000377).

### Main Effect of Nitroglycerin Treatment on Gene Expression

Differential gene expression in response to NTG treatment across studied nervous system regions (main effect of NTG) was investigated. The 11 genes exhibiting the highest NTG effect are listed in Table 5. An expanded list of 165 genes differentially expressed between the NTG and CON groups including the previous genes is provided in Table S5. The majority of these differentially expressed genes (86%) were over-expressed in NTG-treated relative to CON mice. Among the genes with the highest over-expression in NTG–treated compared to CON mice were: Aldehyde dehydrogenase 1 family member A1 (*Aldh1a1*); cytotoxic T lymphocyte-associated protein 2 beta (*Ctla2b*); solute carrier family 7 cationic amino acid transporter, y+ (*Slc7a2*); circadian rhythm period 3 gene (*Per3*); inositol 1,4,5-trisphosphate receptor type 2 (*Itpr2*); and non-coding RNA sequences including *A930013F10Rik, 1700024F13Rik, 4930572G02Rik*, and *E030003E18Rik* (Table 5, Table S5).

**Table 5:** Genes exhibiting significant *P*-value < 1.5E-04 or FDR-adjusted *P*-value < 2.9E-01differential expression between nitroglycerin-treated and control mice.

**Gene symbol**	**Gene name**	**NTG-CON^a^**	***P*-value**	**FDR *P*-value^b^**
*Gm32234*	Uncharacterized protein Gm32234	1.02	5.2E-06	1.1E-01
*Aldh1a1*	Retinal dehydrogenase 1	0.49	2.1E-05	2.3E-01
*Ctla2b*	Protein CTLA-2-beta	0.54	3.4E-05	2.5E-01
*Gm33697*	Predicted gene, 33697 (Gm33697)	0.71	6.0E-05	2.9E-01
*Ttll4*	Tubulin polyglutamylase TTLL4	0.15	9.1E-05	2.9E-01
*Per3*	Period circadian protein homolog 3	0.22	9.5E-05	2.9E-01
*Itpr2*	Inositol 1,4,5-trisphosphate receptor type 2	0.27	1.0E-04	2.9E-01
*Mfsd9*	Major facilitator superfamily domain-containing protein 9	−0.20	1.1E-04	2.9E-01
*Gm31045*	Predicted gene, 31045 (Gm31045)	−0.77	1.4E-04	2.9E-01
*Ubn2*	Ubinuclein-2	0.12	1.5E-04	2.9E-01
*A930013F10Rik*	RIKEN cDNA A930013F10 gene	0.27	1.5E-04	2.9E-01

a *Log_2_(fold change) between nitroglycerin (NTG)-treated relative to control (CON) mice*.

b *False discovery rate (FDR) adjusted P-value*.

### Functional Analysis of the Genes Exhibiting Nitroglycerin Treatment Effects

The most significant DAVID clusters of functional categories enriched among the genes differentially expressed across studied nervous system regions of NTG-treated and CON mice are presented in Table 6. The extended list of DAVID clusters of enriched categories among the differentially expressed genes in NTG-treated relative to CON mice among the over- and under-expressed genes in NTG-treated relative to CON mice is presented in the Table S6. Informative clusters of enriched categories include the terms: glutamatergic synapse and dopaminergic synapse (KEGG mmu04724 and mmu04728), the rhythmic process (GO:0048511), protein phosphorylation (GO:0018108), ATP binding (GO:0005524), and kinase activity (GO:0004672), and ion transport processes (GO:0006811).

**Table 6:** Clusters of Gene Ontology (GO) categories enriched (DAVID enrichment score ES > 2.0) among genes differentially expressed between nitroglycerin-treated and control mice across studied nervous system regions.

**Category^a^**	**GO identifier**	**GO name**	***P*-value**	**FDR *P*-value^b^**
**Cluster 1 (ES: 2.2)**				
MF	GO:0004713	Protein tyrosine kinase activity	1.6E-04	4.1E-02
BP	GO:0018108	Peptidyl-tyrosine phosphorylation	1.7E-04	1.3E-01
MF	GO:0004672	Protein kinase activity	4.0E-04	9.8E-02
BP	GO:0006468	Protein phosphorylation	7.4E-04	4.6E-01
BP	GO:0046777	Protein autophosphorylation	1.1E-03	6.0E-01
BP	GO:0016310	Phosphorylation	1.1E-03	6.1E-01
MF	GO:0005524	ATP binding	4.0E-03	6.5E-01
**Cluster 2 (ES: 2.1)**				
KEGG	mmu04724	Glutamatergic synapse	1.5E-04	1.8E-02
BP	GO:0048511	Rhythmic process	2.2E-03	8.3E-01
KEGG	mmu04728	Dopaminergic synapse	3.0E-03	3.0E-01
**Cluster 3 (ES: 2.0)**				
BP	GO:0006811	Ion transport	3.4E-03	9.4E-01
MF	GO:0005216	Ion channel activity	5.9E-03	7.8E-01

a *BP, biological process; MF, molecular function; KEGG, KEGG pathway*.

b *False discovery rate (FDR) adjusted P-value*.

Results from the GSEA functional analysis within genes over- and under-expressed in NTG-treated relative to CON mice offered complementary insights into the impact of chronic NTG treatment. Table 7 lists the most significantly enriched (*P*-value < 0.0005; normalized enrichment score > |1.8|) and relevant functional categories, selected to minimize redundancies and the corresponding Table S7 presents the extended and complete lists of categories uncovered by GSEA. Nucleoside binding was enriched among the genes under-expressed in NTG-treated mice across both nervous system regions (Table 7). Conversely, anion transmembrane transport (GO:0098656) was significantly enriched among the genes over-expressed in NTG-treated relative to CON mice. The GSEA analysis was able to narrow down this transport to L-alpha-amino acid (GO:0015807) and L-glutamic acid is an important source of GABA.

**Table 7:** Gene Ontology (GO) categories enriched among genes differentially expressed between nitroglycerin-treated and control mice across studied nervous system regions.

**GO category^a^**	**GO identifier**	**GO name**	**NES^b^**	***P*-value**	**FDR *P*-value^c^**
**UNDER-EXPRESSED IN NITROGLYCERIN-TREATED RELATIVE TO CONTROL MICE**					
MF	GO:0001882	Nucleoside binding	−1.92	0.0E+00	2.3E-02
MF	GO:0035639	Purine ribonucleoside triphosphate binding	−1.88	0.0E+00	3.4E-02
**OVER-EXPRESSED IN NITROGLYCERIN-TREATED RELATIVE TO CONTROL MICE**					
BP	GO:0003333	Amino acid transmembrane transport	1.81	1.7E-03	1.9E-02
MF	GO:0046943	Carboxylic acid transmembrane transporter activity	1.81	0.0E+00	2.0E-02
MF	GO:0022804	Active transmembrane transporter activity	1.81	0.0E+00	2.2E-02
MF	GO:0008509	Anion transmembrane transporter activity	1.84	0.0E+00	1.5E-02
BP	GO:0098656	Anion transmembrane transport	1.84	0.0E+00	1.6E-02
BP	GO:0006865	Amino acid transport	1.84	0.0E+00	1.7E-02
MF	GO:0008514	Organic anion transmembrane transporter activity	1.89	0.0E+00	4.3E-03
BP	GO:1902475	L alpha amino acid transmembrane transport	1.92	0.0E+00	1.5E-03
MF	GO:0015179	L amino acid transmembrane transporter activity	1.94	0.0E+00	1.2E-03
BP	GO:0015807	L amino acid transport	2.00	0.0E+00	0.0E+00

a *BP, biological process; MF, molecular function*.

b *Normalized enrichment score where positive and negative value refer to over- and under-expressed in nitroglycerin-treated relative to control mice, respectively*.

c *False discovery rate (FDR) adjusted P-value*.

### Main Effect of Nervous System Region on Gene Expression

The focus of this study is understanding the disruption in transcriptome by the hyperalgesia-evoking NTG treatment in both or either TG and NA. The comparison of gene expression between nervous system regions is provided as molecular confirmation of the regions studied and for completeness. Differences in gene expression between regions were, *per se*, expected.

Supporting the different roles of both investigated regions, 361 genes were differentially expressed at *P*-value < 5.0 E-10 and log fold change > |4| (Table S8) and 37% of these genes were over-expressed in the TG relative to NAc. Table 8 lists the most differentially expressed genes between both nervous system regions. An 8-fold over-expression of the migraine susceptibility gene potassium two pore domain channel subfamily K member 18 (*Kcnk18*) in TG relative to NA (*P*-value < 5.0 E-10) was detected.

**Table 8:** Top 20 most differentially expressed genes (*P*-value < 1.0E-10) between the trigeminal ganglia (TG) and the nucleus accumbens (NAc).

**Gene symbol**	**Gene name**	**TG-NAc^a^**
**OVER-EXPRESSED IN NITROGLYCERIN-TREATED RELATIVE TO CONTROL MICE**		
*Mrgprd*	MAS-related GPR, member D	9.74
*Mpz*	Myelin protein zero	9.51
*Isl2*	Insulin related protein 2	9.43
*Prph*	Peripherin	9.37
*Acpp*	Acid phosphatase, prostate	9.23
*Tmem233*	Transmembrane protein 233	9.22
*Gfra3*	Glial cell line derived neurotrophic factor family receptor alpha 3	9.21
*Tusc5*	Tumor suppressor candidate 5	9.11
*Scn10a*	Sodium channel, voltage-gated, type X, alpha	9.10
*Pirt*	Phosphoinositide-interacting regulator of transient receptor potential channels	8.99
*Ahnak2*	AHNAK nucleoprotein 2	8.97
*D130009I18Rik*	RIKEN cDNA D130009I18 gene	8.93
*Pou4f1*	POU domain, class 4, transcription factor 1	8.90
*Trappc3l*	Trafficking protein particle complex 3 like	8.83
*Calca*	Calcitonin/calcitonin-related polypeptide, alpha	8.78
*Ppp1r1c*	Protein phosphatase 1, regulatory (inhibitor) subunit 1C	8.70
*Avil*	Advillin	8.63
*Wdr72*	WD repeat domain 72	8.63
*Scn11a*	Sodium channel, voltage-gated, type XI, alpha	8.55
*Prx*	Thioredoxin peroxidase, pseudogene 1	8.47
**UNDER-EXPRESSED IN NITROGLYCERIN-TREATED RELATIVE TO CONTROL MICE**		
*Ankrd63*	Ankyrin repeat domain 63	−9.44
*Hpcal4*	Hippocalcin-like 4	−8.41
*Drd1*	Dopamine receptor D1	−8.15
*Dlx6os1*	Distal-less homeobox 6, opposite strand 1	−7.94
*Foxg1*	Forkhead box G1	−7.92
*Gpr88*	G-protein coupled receptor 88	−7.90
*Otof*	Otoferlin	−7.88
*Icam5*	Intercellular adhesion molecule 5, telencephalin	−7.77
*Serpina9*	Serine (or cysteine) peptidase inhibitor, clade A (alpha-1 antiproteinase, antitrypsin), member 9	−7.64
*Camkv*	CaM kinase-like vesicle-associated	−7.45
*Npy*	Neuropeptide Y	−7.45
*Rasal1*	RAS protein activator like 1 (GAP1 like)	−7.44
*Cecr6*	Cat eye syndrome chromosome region, candidate 6	−7.29
*Itpka*	Inositol 1,4,5-trisphosphate 3-kinase A	−7.24
*Vipr1*	Vasoactive intestinal peptide receptor 1	−7.20
*Zfp831*	Zinc finger protein 831	−7.16
*Gpr6*	G protein-coupled receptor 6	−7.10
*Grm5*	G protein coupled receptor, family C, group 1, member E	−7.07
*Emx2*	Empty spiracles homeobox 2	−6.80
*Dlgap2*	Discs, large (Drosophila) homolog-associated protein 2	−6.74

a *Log_2_(fold change) between TG and NAc*.

Table 9 presents the most significantly enriched DAVID clusters (enrichment score > |1.7|) of selected and relevant enriched categories, and the corresponding Table S9 presents the extended list of these categories. The most enriched categories within the top clusters include: ion transport channel activity (GO:0005216; enrichment score = 6); KEGG pathway neuroactive ligand-receptor interaction (mmu04080; enrichment score = 5.3); and KEGG pathway endocannabinoid signaling (mmu04723; enrichment score = 3.1).

**Table 9:** Clusters of enriched functional categories (DAVID enrichment score ES > 1.7) among the genes differentially expressed between the trigeminal ganglia and nucleus accumbens.

**Category^a^**	**GO identifier**	**GO name**	***P*-value**	**FDR *P*-value^b^**
**Cluster 1 (ES: 6)**				
MF	GO:0005216	Ion channel activity	4.7E-14	2.1E-11
BP	GO:0006813	Potassium ion transport	7.1E-06	1.2E-03
**Cluster 2 (ES: 5.3)**				
KEGG	mmu04080	Neuroactive ligand-receptor interaction	4.0E-12	5.5E-10
**Cluster 3 (ES: 5.1)**				
BP	GO:0019228	Neuronal action potential	2.5E-09	1.1E-06
MF	GO:0005272	Sodium channel activity	1.1E-04	7.9E-03
**Cluster 4 (ES: 3.1)**				
KEGG	mmu04723	Retrograde endocannabinoid signaling	2.4E-05	1.1E-03
KEGG	mmu05032	Morphine addiction	7.6E-05	2.6E-03
MF	GO:0004890	GABA-A receptor activity	3.3E-03	9.4E-02
**Cluster 5 (ES: 2.4)**				
MF	GO:0005262	Calcium channel activity	4.4E-04	1.9E-02
**Cluster 6 (ES: 2.2)**				
BP	GO:0033693	Neurofilament bundle assembly	7.8E-04	5.1E-02
**Cluster 7 (ES: 2.1)**				
KEGG	mmu05030	Cocaine addiction	8.8E-05	2.4E-03
KEGG	mmu05031	Amphetamine addiction	3.4E-03	3.1E-02
**Cluster 8 (ES: 1.8)**				
BP	GO:0019226	Transmission of nerve impulse	3.9E-04	3.1E-02
**Cluster 9 (ES: 1.7)**				
KEGG	mmu04728	Dopaminergic synapse	9.3E-04	1.4E-02
KEGG	mmu04724	Glutamatergic synapse	1.7E-03	2.0E-02
KEGG	mmu04713	Circadian entrainment	3.6E-03	3.1E-02

a *BP, biological process; MF, molecular function; KEGG, KEGG pathway*.

b *False discovery rate (FDR) adjusted P-value*.

### Regulatory Networks

Regulatory network analysis aided in the identification of transcription factors that target many of the genes dysregulated by NTG treatment. Table 10 lists the enriched transcription factors corresponding to three complementary lists of differentially expressed genes (*P*-value < 0.005) between the NTG and CON groups. These lists represented differential expression within TG (338 genes), within NAc (103 genes), and across both regions (165 genes). Enriched transcription factors in the TG list included: CCAAT/enhancer binding protein alpha (*Cebpa*; normalized enrichment score = 5.2) and small muscle protein, X-linked (*Smpx*; normalized enrichment score = 4.4). Enriched transcription factors in the NAc list included: direct IAP-binding protein with low PI (*Diablo*; normalized enrichment score = 4.81) and yin and yang 1 (*Yy1*; normalized enrichment score = 4.34). Enriched transcription factors across regions included: E2F transcription factor 1 (*E2f1*; normalized enrichment score = 5.1), BarH-like homeobox 1 (*Barx1*; normalized enrichment score = 4.5), transcription factor GATA binding protein 3 (*Gata3*), and NK2 homeobox 2 (*Nkx2-2*).

**Table 10:** Transcription factors (TFs) enriched (iRegulon normalized enrichment score NES > 3.0) among the genes differentially expressed between nitroglycerin-treated and control mice within the trigeminal ganglia (TG, target gene number = 338), within the nucleus accumbens (NAc, target gene number = 103), and across both nervous system regions (target gene number = 165).

**TF**	**NES**	**Target gene *N***
**TG**		
*Cebpa*	5.186	18
*Smpx*	4.447	129
*Smad1*	4.185	36
*Snrnp70*	3.94	25
*Sp1*	3.896	160
*Rfx2*	3.749	103
*Tfap4*	3.689	83
*Pax6*	3.626	33
*Atf1*	3.216	18
*Tead3*	3.197	15
*Pax3*	3.191	21
**NAc**		
*Diablo*	4.81	16
*Yy1*	4.347	22
*Six6*	4.268	24
*Dmrtc2*	3.902	11
*Tcf4*	3.871	10
*Tbx5*	3.851	8
*Pura*	3.837	7
*Rfx2*	3.74	15
*Hesx1*	3.455	17
*Gata3*	3.435	10
*Pou2f1*	3.431	10
*Runx3*	3.315	14
*Ahr*	3.275	21
*Mafa*	3.073	10
**ACROSS REGIONS**		
*E2f1*	5.128	16
*Barx1*	4.483	18
*Mnt*	3.997	15
*Hlf*	3.683	21
*Tead1*	3.568	24
*Gata3*	3.553	28
*Zfp706*	3.51	19
*Kdm4d*	3.491	19
*Pou5f1*	3.446	40
*Nf1*	3.411	18
*Pura*	3.329	10
*Nkx2-2*	3.241	14
*Jun*	3.209	7
*Pou3f3*	3.157	21
*Stat1*	3.15	26
*Hoxa13*	3.029	11

**Figure 2:**
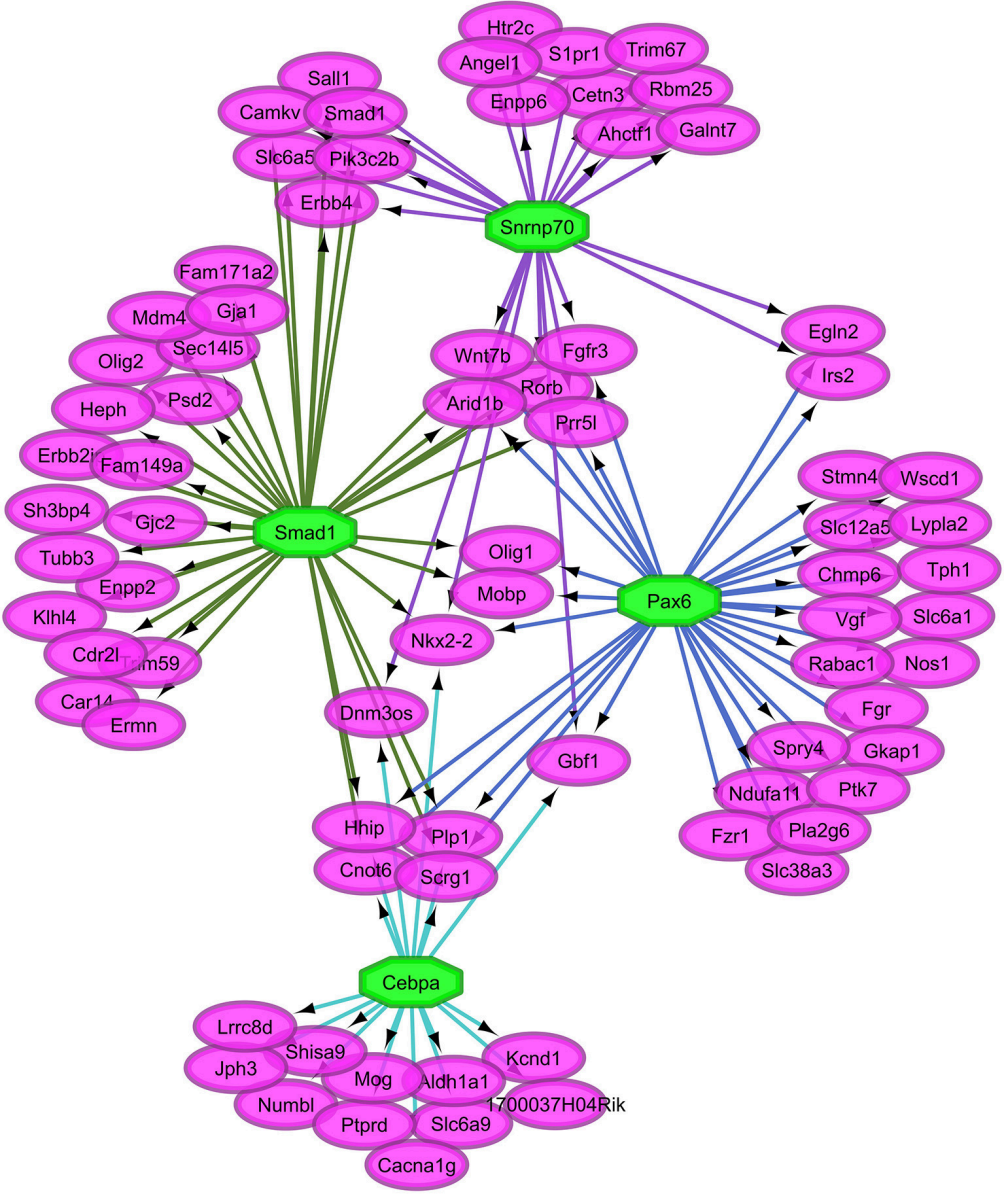
Relationship between the transcription factors (octagons) most enriched (normalized enrichment score >3.5 and <40 genes to facilitate visualization) among genes (ovals) differentially expressed between nitroglycerin-treated and control mice within the trigeminal ganglia.

**Figure 3:**
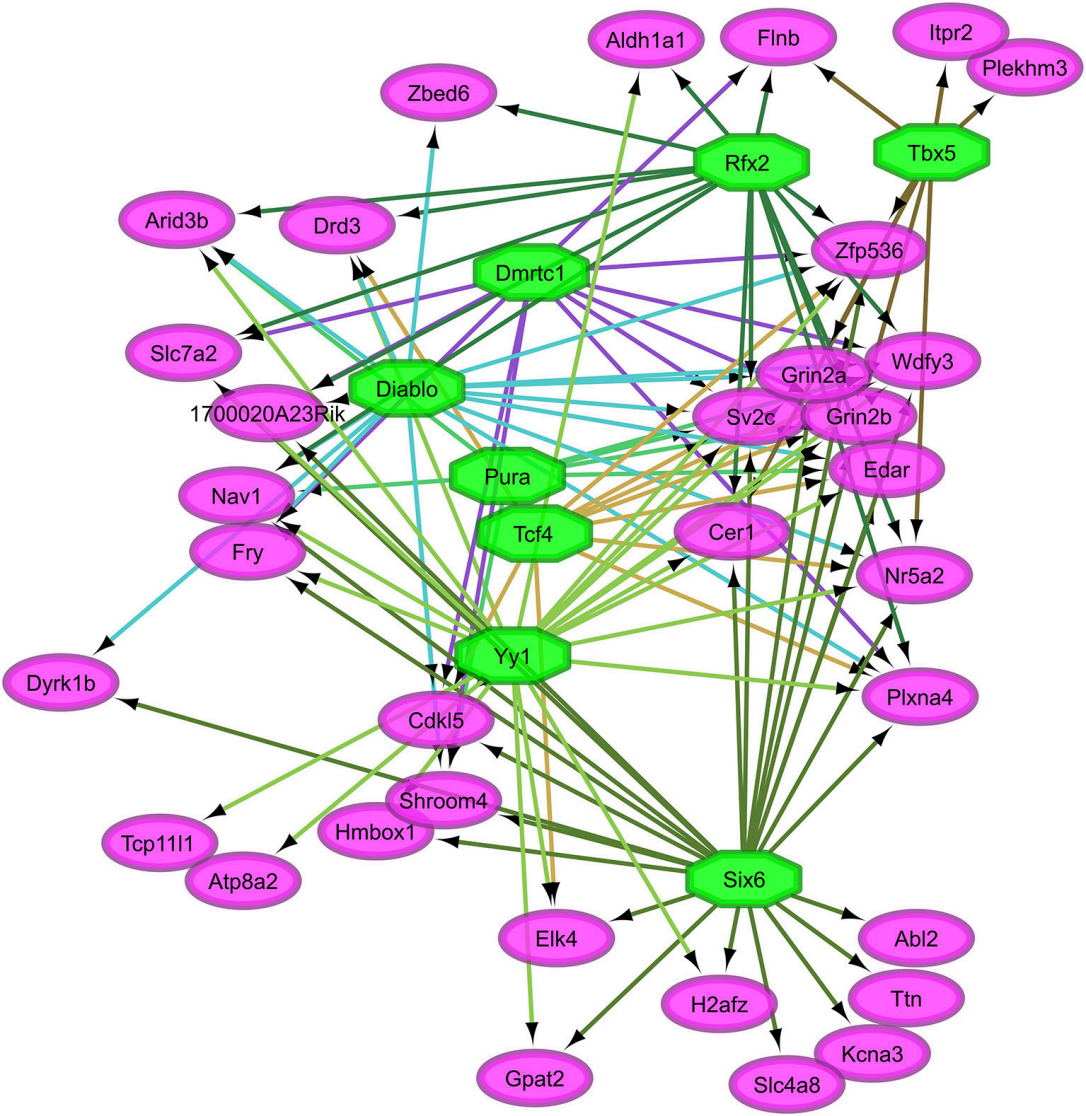
Relationship between the transcription factors (octagons) most enriched (normalized enrichment score >3.5 and <40 genes to facilitate visualization) among genes (ovals) differentially expressed between nitroglycerin-treated and control mice within the nucleus accumbens.

**Figure 4:**
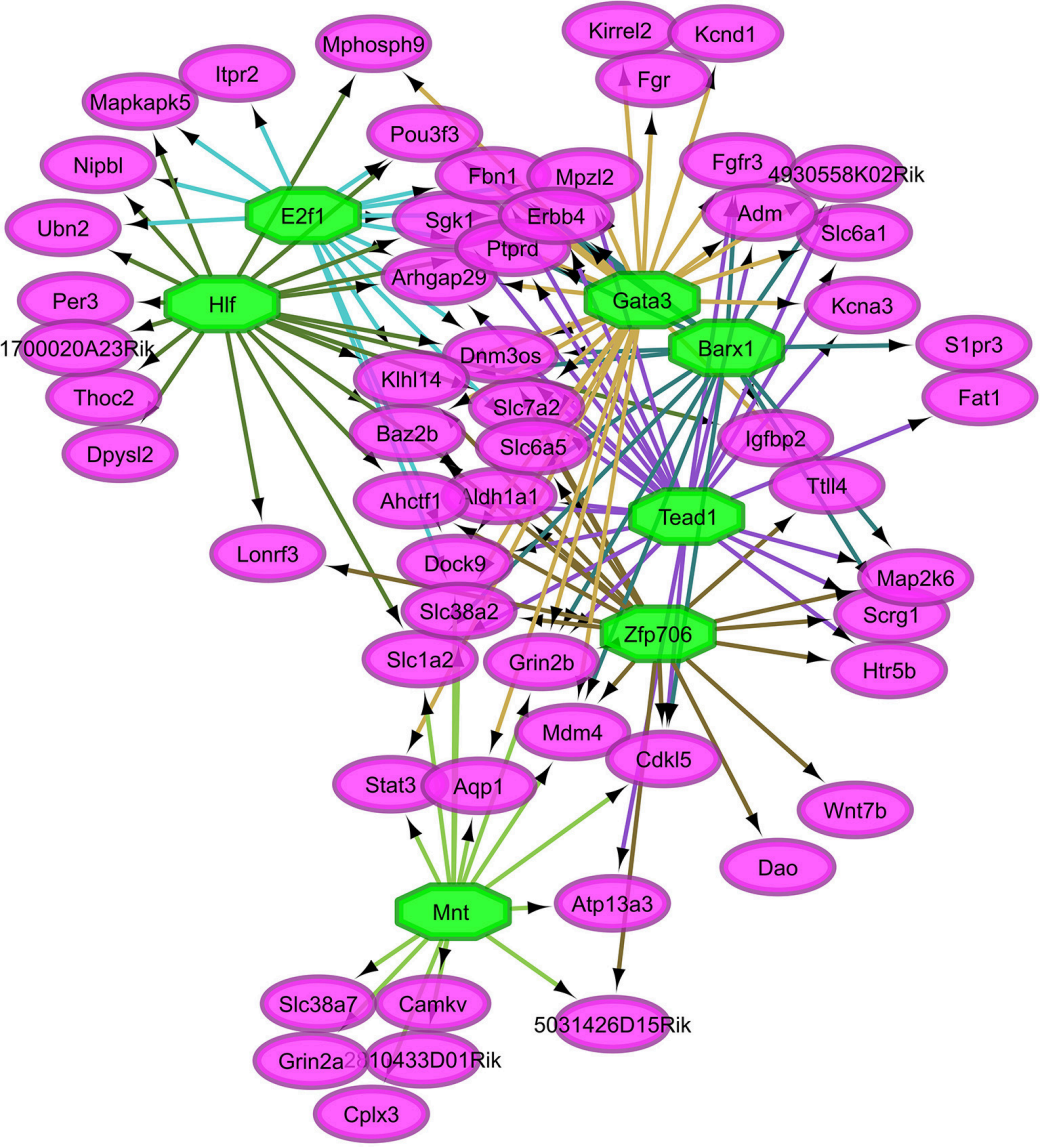
Relationship between the transcription factors (octagons) most enriched (normalized enrichment score >3.5 and <40 genes to facilitate visualization) among genes (ovals) differentially expressed between nitroglycerin-treated and control mice.

Overall, our study of regulatory networks impacted by NTG treatment confirmed our finding (Table 2) related to the important role of transcriptional regulation on the chronic NTG model studied. Figures 2, 3 depict the relationship between the transcription factors (octagons) most enriched (normalized enrichment score >3.5 and <40 genes to facilitate visualization) among genes (ovals) differentially expressed between NTG-treated and CON mice within the TG and NAc, respectively. Figure 4 depicts the relationship between the transcriptional factors most enriched (normalized enrichment score >3.5 and <40 genes) among the genes exhibiting a NTG treatment effect. The thresholds were used to facilitate the visualization of relationships between transcription factors and between transcription factors and their target genes. Another notable finding is that the transcription factors in the regulatory network disrupted by NTG in the TG (Figure 2) are less connected by common target genes than the regulatory network disrupted by NTG in the NAc (Figure 3) and across nervous system regions (Figure 4).

The TG regulatory network disrupted by NTG depicted in Figure 2 highlights the connection between all four enriched transcription factors through other transcription factors that were differentially expressed. Transcription factors *Snrnp70, Smad1, Pax6*, and *Cebpa* are connected through transcription factors oligodendrocyte transcription factor 1 (*Olig1*) and *Nkx2-2*. Transcription factor genes *Olig1* and *Nk2-2* were over-expressed in NTG-treated relative to CON mice in the TG (*P*-value < 0.0032 and < 0.026 respectively, Table S1). Several genes targets of the enriched transcription factors in the TG exhibited significant treatment-by-region effect (Table 1, Table S1) including: CaM kinase-like vesicle-associated protein (*Camkv*), egl-9 family hypoxia-inducible factor 2 (*Egln2*), WSC domain containing protein 1 (*Wscd1*), and stathmin 4 (*Stmn4*).

Figure 3 highlights the interconnection between transcription factors enriched among genes dysregulated by NTG in the NAc. Among these transcription factors are: Sine oculis-related homeobox 6 (*Six6*), doublesex- and mab-3-related transcription factor C1 (*Dmrtc1*), and regulatory factor X, 2 influences HLA class II expression (*Rfx2*). A large number of target genes shown in Figure 4 were among the most differentially expressed genes between NTG-treated and CON mice listed in Table 5 and in Table S5 including: *Aldh1a1, Per3, Slc7a2, Htr5b*, sphingosine 1-phosphate receptor 3 (*S1pr3*), ubinuclein-2 (*Ubn2*), tubulin polyglutamylase (*Ttll4*), and inositol 1,4,5-trisphosphate receptor type 2 (*Itpr2*). This result highlights the critical role of transcription factors in the dysregulation of genes associated with NTG-treatment.

## Discussion

The findings from the pain threshold experiment confirmed that the chronic intermittent NTG treatment used in this study triggered mechanical hyperalgesia in mouse population studied. The NO donor NTG has been used extensively to understand the nociceptive system and pain processing (Cury et al., 2011). Nitroglycerin reliably triggers headache in normal subjects, and migraine without aura in migraine susceptible patients (Iversen et al., 1989; Christiansen et al., 1999; Afridi et al., 2005); and NTG-evoked migraine is a commonly used experimental paradigm in humans (Olesen, 2008, 2010). Nitroglycerin-evoked hyperalgesia in rodents has been developed as a model for sensory hypersensitivity associated with migraine (Bates et al., 2010; Markovics et al., 2012). Acute NTG treatment was previously shown to produce thermal and mechanical allodynia in mice that was reversed by the anti-migraine therapies sumatriptan (Bates et al., 2010; Markovics et al., 2012), and a calcitonin-gene-related peptide receptor antagonist (Capuano et al., 2014). In addition, in a transgenic mouse model of familial migraine, animals expressing a human migraine gene (casein kinase 1 delta) showed an even greater sensitivity to NTG-evoked hyperalgesia (Brennan et al., 2013). Further, NTG has also been shown to produce light-aversive behavior (Markovics et al., 2012), and increased meningeal blood flow in mice (Greco et al., 2011; Markovics et al., 2012); two hallmark characteristics of migraine.

Expanding upon the acute NTG model, we have developed a model of chronic migraine-associated pain. Figure 1, depicts the enhanced peripheral and cephalic hypersensitivity observed in the NTG mouse group. Chronic intermittent NTG produces a long-lasting and severe basal hyperalgesia, which is sensitive to migraine preventive treatment. This model has been extensively characterized pharmacologically and we have used it to identify novel therapeutic targets for migraine (Pradhan et al., 2014b; Tipton et al., 2016; Moye and Pradhan, 2017), as well as to identify mechanisms that can lead to migraine chronification (Ben Aissa et al., 2017).

Both, the NAc and TG have been associated with pain perception (Akerman et al., 2011; Schwartz et al., 2014). The present study of these regions in mice displaying hyperalgesia evoked by NTG treatment enabled to investigate molecular players that are either distinctly or similarly impacted by this treatment. Our results offer novel insights into the nervous system region-dependent and region-independent molecular mechanisms associated with NTG-evoked chronic hyperalgesia. These findings can help in the identification of solutions to ameliorated chronic pain such as migraine.

### Genes and Processes Affected by Nitroglycerin Treatment in a Region-Specific Manner

Among the genes exhibiting significant treatment-by-nervous system region interaction effects, the enrichment of positive regulation of transcription processes (Table 2, Table S2) is consistent with the established activation of the transcription factor *NF-kB* in migraine studies (Reuter et al., 2002; Greco et al., 2005). Likewise, the enrichment of T cell-mediated cytotoxicity in the TG (Tables 3, 4) was also reported in the TG of rats exposed to acute NTG treatment (Pedersen et al., 2016). The genes annotated to this functional category include *Lrrc8a, Efnb*, and *Siglech* listed in Table 1, Table S1.

Several of the genes exhibiting region-specific NTG effect have been associated with migraine in humans. The activity of the volume regulated anion channel protein coded by *Lrrc8* has been associated with spreading through the brain focal depolarization of neurons and glial cells that occur in humans and animal models during migraines (Mongin, 2016). Susceptibility for migraine loci on *Wscd1* (Table S1) were identified on a large meta-analysis in humans (Gormley et al., 2016). TIMP Metallopeptidase Inhibitor 4 (*Timp4*), an inhibitor of the matrix metalloproteinases, has been linked to the pathophysiology of migraine (Bernecker et al., 2011).

The unexpected enrichment of the categories sensory perception of light stimuli and retina process among the genes over-expressed in NTG-treated compared to CON mice was elucidated by the evaluation of the genes supporting this finding (Tables 3, 4). Genes supporting these enrichments included calcium channel, voltage-dependent, alpha 2/delta subunit 4 (*Cacna2d4*), RAR-related orphan receptor beta (*Rorb*) and phosphodiesterase 6G, cGMP-specific, rod, gamma (*Pde6g*). These genes are all associated with pain and migraine (Schleithoff et al., 1999; D'Souza et al., 2008; Descalzi et al., 2017), in addition to light stimuli, thus explaining the enrichment of the latter categories.

The significant treatment-by-region effect on *Slc32a1* (Table 1) and *ErbB4* (Table S1) support the enrichment of GABA-related pathways. *Slc32a1* is used as a marker for GABAergic neurons that modulate transmission of pain-related signal (Du et al., 2017). *ErbB4* is mainly expressed in the GABAergic interneurons and is a receptor of neuregulin which is associated with neuropathic pain (Yau et al., 2003). Our findings offer insights into the molecular mechanism that are the target of effective migraine therapies such as anticonvulsants, topiramate, and valproic acid that disrupt the GABA pathway (Johannessen and Johannessen, 2003; Calabresi et al., 2007; Mulleners and Chronicle, 2008).

In addition to the GABA pathway, *Slc32a1* is mapped to the morphine addiction, nicotine addiction, and endocannabinoid signaling pathways. Likewise, Penk-derived opioid neuropeptides are known to influence chronic tension-type headaches (Langemark et al., 1995; Packard and Ham, 1997). These findings uncover intersectionality between the molecular mechanisms disrupted by NTG-treatment that evoke hyperalgesia and by drugs of abuse.

An interesting finding is that NTG treatment was associated both with lower expression of genes involved in innate immune response in the NAc and with higher expression of genes involved in adaptive immune response in the TG (Table 4). This association is in agreement with reports of the role of the trigeminovascular system, neuropeptides, and inflammatory cytokines in the pathophysiology of migraine (Farajdokht et al., 2017).

The glutamate receptor signaling pathway was enriched among the genes over-expressed in the NAc of NTG-treated mice. This result is in agreement with reports of association between migraine and increased glutamate transmission (Sarchielli et al., 2007; van Dongen et al., 2017). This enrichment is supported by the significant treatment-by-region interaction on *Slc1a2* (Table S1), a gene that has nucleotide variants associated with migraine symptoms (García-Martín et al., 2014). The expression of *Opalin*, a gene that codes for a component of myelin, exhibited treatment-by-region interaction effect (Table 1). This profile corroborates the enrichment of myelin formation among genes implicated in migraine pain signaling (Eising et al., 2016).

The regulation of reactive oxygen species process was enriched among the genes under-expressed in the NAc of NTG-treated relative to CON mice (Table 4). The under-expression of these genes including the oxygen sensor *Egln2* (Table S1) may elevate the level of reactive oxygen species that have been associated with migraine (Quaegebeur et al., 2016). An alternative mechanism that may trigger hyperalgesia in NTG-treated mice may be associated with the hormone *Nppc* that exhibited a significant treatment-by-region interaction effect (Table S1). *Nppc* can produce natriuretic peptides and has been associated with hemiplegic migraine (Marchenkova et al., 2016a,b).

### Genes and Processes Affected by Nitroglycerin Treatment in a Region-Independent Manner

The enrichment of dopaminergic synapses pathway among genes differentially expressed between NTG and CON mice consistently across regions (Table 6) agrees with indications that disrupted levels of the neurotransmitters such as dopamine and noradrenaline in the corresponding synapse clefts of the pain matrix could evoke in the trigeminal system the release of calcitonin gene-related peptide, a peptide involved in the transmission of migraine pain (D'Andrea and Leon, 2010). This process can be followed by build-up of inflammatory mediators and sensitization of the TG leading to migraine (D'Andrea and Leon, 2010). Dopamine transporters has been associated with migraine and accompanying drug abuse (Cevoli et al., 2006), further reinforcing the link between NTG and drug abuse-triggered hyperalgesia and migraine. *Aldh1a1* and *Itpr2*, both associated with the dopaminergic pathway, were over-expressed between NTG and CON mice (Table 5). Over-expression of *IItpr2* has been associated with induced visceral inflammatory hypersensitivity (Qian et al., 2013) and with neuropathic pain in rats (Maratou et al., 2009). Also over-expressed in NTG, the dysregulation of *Ctla2b* (Table 5) supports the proposal to use cathepsin inhibitors as antihyperalgesics (Irie et al., 2008).

Our findings of under-expressed serotonin receptor and phosphorylation and kinase enrichment in NTG relative to CON mice (Table 6) can be related to reports that serotonin depletion in rats increases nociception-evoked and kinase enabled phosphorylation in the TG (Maneepak et al., 2009). Enrichment of ion transport and phosphorylation processes among genes differentially expressed between NTG-treated and CON mice have been reported in studies of hemiplegic migraine (Ophoff et al., 1996; De Fusco et al., 2003; Dichgans et al., 2005; Schack et al., 2012; Pietrobon, 2013).

The enrichment of the nucleoside binding function among the genes under-expressed in NTG-treated relative to CON mice (Table 7) supports the use of modulators of G protein-coupled receptors that have nucleoside binding capabilities to treat pain and migraine (Venkatakrishnan et al., 2013). The enrichment of the amino acid transport process (Table 7) is supported by the dysregulation of *Slc7a2* (Table 5). The arginine transport facilitated by Slc7a2 can be associated with migraine because arginine is a chemical precursor to NO. Likewise, *Aldh1a1* was dysregulated by NTG and aldehyde dehydrogenases catalyze the conversion of NTG into NO (Sydow et al., 2004). Nitric oxide acts as a vasodilator and triggers headaches whereas inhibition of NO synthase is an effective treatment to relieve migraine attacks (Olesen, 2008). The 8-fold over-expression of the migraine susceptibility gene *Kcnk18* in TG relative to NA (*P*-value < 5.0 E-10) is consistent with reports of over-expression of this gene in the neural-enriched human trigeminal samples (LaPaglia et al., 2018).

The enrichment of rhythmic processes among genes dysregulated by NTG treatment (Table 6) is supported by the over-expression of *Per3* in NTG relative to CON mice (Table 5). *Per3* has been associated with cluster headache (Ofte et al., 2013) and dysregulation of circadian rhythm has been associated with migraine (Solomon, 1991; Hering and Kuritzky, 1992; Pringsheim, 2002). Non-coding RNA sequences including *A930013F10Rik* and *1700024F13Rik* (Table 5) and *4930572G02Rik* and *E030003E18Rik* (Table S5) were over-expressed in NTG-treated relative to CON mice. Non-coding RNAs that regulate glutamate transporters have been proposed for migraine therapy (Gasparini et al., 2016).

### Regulatory Networks

Regulatory network analysis of gene expression disruptions triggered by NTG offers insights into the role of transcription factors and their target genes on chronic hyperalgesia (Table 10). The transcription factors *Cebpa* and *Smpx* were enriched among the genes dysregulated in the TG of NTG-treated mice (Table 10). *Cebpa* has been associated with attenuation of pain hypersensitivity in the spinal cord of mice (Jiang et al., 2017). In mice, *Smpx* is dysregulated by disruption of neurolysin, a molecule implicated in two processes associated with hyperalgesia: pain control and blood pressure regulation (Cavalcanti et al., 2014). The lower level of interconnection among transcription factors and targets genes observed in the TG network (Figure 2) suggests that many therapeutic targets may be needed to effectively revert the network disruption triggered by NTG and associated with chronic hyperalgesia.

A notable finding in the TG regulatory network disrupted by NTG (Figure 2) is that all four enriched transcription factors (*Snrnp70, Smad1, Pax6*, and *Cebpa*) are related through other transcription factors. For example, *Olig1* can have an inhibitory effect on GABAergic interneurons that attenuate neuropathic pain in rodents (Silbereis et al., 2014). Also *Nkx2-2* is expressed in cells that give rise to serotonergic neurons and the modulation of these neurons has been implicated in the sensation of pain (Pattyn et al., 2004).

The transcription factors *Diablo, Six6, Rfx2*, and *Yy1* were enriched among the genes dysregulated by NTG in the NAc (Table 10, Figure 3). Our results support reports that *Diablo* is elevated in rats treated with alkaloid compounds that have been linked to migraine events associated with ischemic stroke (Alsharafi et al., 2015). *Yy1* was enriched among inflammatory pathway genes dysregulated in a transgenic mouse model of migraine (Eising et al., 2017). *Six6* is associated with a hereditary disorder of the eye that presents with migraine symptoms (Burdon et al., 2015) and decreased expression of *Rfx2* elicited increased sensitivity in a model of neuropathic pain (Wheeler et al., 2013). *Six6* is a transcription factor for H2A histone family member Z (*H2afz*) that has been associated with the effects of abused substances (Vadasz et al., 2007; McBride et al., 2009) and migraine is one of these symptoms (Granella et al., 1987).

Disruption of NF-κb levels has been associated with migraine (Karatas et al., 2013) and acute nitroglycerin treatment in rats (Greco et al., 2005). In our study, transcription factor targets of NF-κb including *Sox9* and *Ahctf1* were enriched or differentially expressed whereas only NF-κb p100 subunit (*Nfkb2*) was over-expressed in NTG-treated relative to CON mice. This observation suggests that NF-κb may initiate pain whereas downstream molecular targets may maintain the long-lasting molecular dysregulation responsible for chronic pain sensation.

## Conclusions

Comparison of the NTG and CON groups confirmed that chronic NTG treatment substantially decreased the mechanical pain threshold in the treated mice. The innovative transcriptome comparison between two central nervous regions offered novel insights into region-dependent and -independent differential gene expression and networks that can be associated with hyperalgesia. The genes that exhibited opposite differential expression in response to NTG across regions included: *Opalin, Efnb3, Slc32a1, Penk, Rorb, Lrrc8a, Cacna1b, H2-Eb2, Gm1673*, and *Egln2*. The genes that exhibited consistent NTG expression pattern, albeit of different magnitudes, across regions included *Pou3f3, Erbb4*, and *Slc1a2*. The genes that exhibited consistent pattern and magnitude of differential expression between NTG and CON groups included: *Aldh1a1, Ctla2b, Slc7a2, Per3*, and *Itpr2*.

The detection of differential abundance in multiple genes reported to be distinctively expressed within the regions studied supports the capacity of the experimental design used to detect molecular differences. Further validation of the differential expression patterns using additional quantitative technologies was prevented by insufficient sample from the tested mice and regions. Partially addressing this situation, our discussion focused on differentially expressed genes that were both supported by enriched functional categories and have been previously associated with allodynia and migraine phenotypes with preference to studies in rodents, NTG treatment and similar regions.

Hyperalgesia and migraine phenotypes, and therefore the underlying molecular mechanisms, are not static but rather tend to be condition-dependent. Migraine affects more females than males and aged than young subjects. Moreover, migraine, depression, and stress tend to be present as comorbidies. The present study focused on comparing the transcriptome of two CNS regions that can participate on these phenotypes in adult male mice. Follow-up studies including the comparison of phenotypes and transcriptome patterns associated with hyperalgesia in females and males, young and aged, and across behavior groups are expected to enhance the understanding of molecular players associated with hyperalgesia and migraine. Likewise, additional studies including migraine interventional therapies (sumatriptan, topiramate, and propranolol) and longitudinal studies including NTG treatment suspension and resumption will offer insights into potential multi-factorial interactions that affect the gene expression profiles associated with hyperalgesia and migraine.

The detected enrichment of positive regulation of transcription processes among the genes exhibiting significant treatment-by-nervous system region interaction effects is consistent with the established activation of the transcription factor NF-kB in migraine studies. Similarly, the enrichment of T cell-mediated cytotoxicity was also reported in the TG of rats exposed to acute NTG treatment. Several of the genes exhibiting region-specific NTG effect have been associated with migraine in humans including *Lrrc8* and *Wscd1*.

The significant enrichment of the GABA-related pathway among genes presenting a treatment-by-region effect offer insights into the molecular mechanism that are the target of effective migraine therapies such as anticonvulsants, topiramate, and valproic acid that disrupt the GABA pathway. The enrichment of the GABA pathway and the differential expression of genes associated with addiction between treatments suggest intersectionality between the molecular mechanisms disrupted by NTG-treatment that evoke hyperalgesia and by drugs of abuse.

A thought-provoking result is simultaneous association of NTG treatment with under-expression of genes involved in innate immune response in the NAc and with over-expression of genes involved in adaptive immune response in the TG. This result confirms the participation of the trigeminovascular system, neuropeptides and inflammatory cytokines in the molecular processes of migraine. The known association between migraine and increased glutamate transmission glutamate receptor signaling pathway was confirmed in our study among the genes over-expressed in the NAc of NTG-treated mice. The enrichment of the process of regulation of reactive oxygen species process among the genes under-expressed in the NAc of NTG-treated relative to CON mice is consistent with reports of correspondence between elevated level of reactive oxygen species and migraine

Regulatory network reconstruction highlighted the widespread role of several transcription factors (including *Snrnp70, Smad1, Pax6, Cebpa*, and *Smpx*) among the NTG-disrupted target genes. Overall, this work provides promising leads to help identify critical mechanisms associated with NTG-evoked hyperalgesia with therapeutic applications toward the modulation and management of chronic pain and migraine.

## Author Contributions

AP, SR-Z, JS: conceptualization of the study and provision of resources; LM, AH, ID, ER, SR: animal and RNA-seq experiments, sample collection, and processing; HJ, BS: analysis; BS: data curation; HJ, BS: software implementation; SR-Z, AP: writing of original manuscript draft; AP, HJ, BS, SR, AH, LM, ER, SR-Z, JS: review and editing of the final manuscript.

### Conflict of Interest Statement

The authors declare that the research was conducted in the absence of any commercial or financial relationships that could be construed as a potential conflict of interest.

## References

[B1] Afridi S. K. Matharu M. S. Lee L. Kaube H. Friston K. J. Frackowiak R. S.. (2005). A PET study exploring the laterality of brainstem activation in migraine using glyceryl trinitrate. Brain 128(Pt 4), 932–939. 10.1093/brain/awh416 15705611

[B2] Akerman S. Holland P. R. Goadsby P. J. (2011). Diencephalic and brainstem mechanisms in migraine. Nat. Rev. Neurosci. 12, 570–584. 10.1038/nrn3057 21931334

[B3] Alsharafi W. A. Bi F.-F. Hu Y.-Q. Mujlli H. M. Xiao B. (2015). Effect of Khat on apoptosis and related gene Smac/DIABLO expression in the cerebral cortex of rats following transient focal ischemia. Environ. Toxicol. Pharmacol. 39, 424–432. 10.1016/j.etap.2014.12.011 25569323

[B4] Andrews S. (2010). FastQC: A Quality Control Tool for High Throughput Sequence Data. Available online at: http://www.bioinformatics.babraham.ac.uk/projects/fastqc Google Scholar.

[B5] Bates E. A. Nikai T. Brennan K. C. Fu Y. H. Charles A. C. Basbaum A. I.. (2010). Sumatriptan alleviates nitroglycerin-induced mechanical and thermal allodynia in mice. Cephalalgia 30, 170–178. 10.1111/j.1468-2982.2009.01864.x 19489890 PMC4854191

[B6] Bellamy J. Bowen E. J. Russo A. F. Durham P. L. (2006). Nitric oxide regulation of calcitonin gene-related peptide gene expression in rat trigeminal ganglia neurons. Eur. J. Neurosci. 23, 2057–2066. 10.1111/j.1460-9568.2006.04742.x 16630053 PMC1486900

[B7] Ben Aissa M. Tipton A. F. Bertels Z. Gandhi R. Moye L. S. Novack M.. (2017). Soluble guanylyl cyclase is a critical regulator of migraine-associated pain. Cephalalgia 38, 1471–1484. 10.1177/0333102417737778 29022756 PMC5916516

[B8] Benjamini Y. Hochberg Y. (1995). Controlling the false discovery rate: a practical and powerful approach to multiple testing. J. R. Stat. Soc. 57, 289–300. 10.1111/j.2517-6161.1995.tb02031.x

[B9] Bernecker C. Pailer S. Kieslinger P. Horejsi R. Möller R. Lechner A.. (2011). Increased matrix metalloproteinase activity is associated with migraine and migraine-related metabolic dysfunctions. Eur. J. Neurol. 18, 571–576. 10.1111/j.1468-1331.2010.03205.x 20825467

[B10] Bigal M. E. Serrano D. Reed M. Lipton R. B. (2008). Chronic migraine in the population: burden, diagnosis, and satisfaction with treatment. Neurology 71, 559–566. 10.1212/01.wnl.0000323925.29520.e7 18711108

[B11] Bray N. L. Pimentel H. Melsted P. Pachter L. (2016). Near-optimal probabilistic RNA-seq quantification. Nat. Biotechnol. 34, 525–527. 10.1038/nbt.3519 27043002

[B12] Brennan K. C. Bates E. A. Shapiro R. E. Zyuzin J. Hallows W. C. Huang Y.. (2013). Casein kinase idelta mutations in familial migraine and advanced sleep phase. Sci. Transl. Med. 5:183ra156. 10.1126/scitranslmed.3005784 23636092 PMC4220792

[B13] Burdon K. P. Mitchell P. Lee A. Healey P. R. White A. J. Rochtchina E.. (2015). Association of open-angle glaucoma loci with incident glaucoma in the Blue Mountains Eye Study. Am. J. Ophthalmol. 159, 31–36.e1. 10.1016/j.ajo.2014.09.020 25242315 PMC4265734

[B14] Burstein R. Jakubowski M. (2005). Unitary hypothesis for multiple triggers of the pain and strain of migraine. J. Comp. Neurol. 493, 9–14. 10.1002/cne.20688 16258903

[B15] Caetano-Anollés K. Mishra S. Rodriguez-Zas S. L. (2015). Synergistic and antagonistic interplay between Myostatin gene expression and physical activity levels on gene expression patterns in *Triceps Brachii* muscles of C57/BL6 mice. PloS ONE 10:e0116828. 10.1371/journal.pone.0116828 25710176 PMC4339580

[B16] Caetano-Anollés K. Rhodes J. S. Garland T. Jr Perez S. D. Hernandez A. G. Southey B. R.. (2016). Cerebellum transcriptome of mice bred for high voluntary activity offers insights into locomotor control and reward-dependent behaviors. PLoS ONE 11:e0167095. 10.1371/journal.pone.0167095 27893846 PMC5125674

[B17] Calabresi P. Cupini L. M. (2005). Medication-overuse headache: similarities with drug addiction. Trends Pharmacol. Sci. 26, 62–68. 10.1016/j.tips.2004.12.008 15681022

[B18] Calabresi P. Galletti F. Rossi C. Sarchielli P. Cupini L. M. (2007). Antiepileptic drugs in migraine: from clinical aspects to cellular mechanisms. Trends Pharmacol. Sci. 28, 188–195. 10.1016/j.tips.2007.02.005 17337068

[B19] Capuano A. Greco M. C. Navarra P. Tringali G. (2014). Correlation between algogenic effects of calcitonin-gene-related peptide (CGRP) and activation of trigeminal vascular system, in an *in vivo* experimental model of nitroglycerin-induced sensitization. Eur. J. Pharmacol. 740, 97–102. 10.1016/j.ejphar.2014.06.046 24998872

[B20] Cavalcanti D. M. Castro L. M. Neto J. C. R. Seelaender M. Neves R. X. Oliveira V.. (2014). Neurolysin knockout mice generation and initial phenotype characterization. J. Biol. Chem. 289, 15426–15440. 10.1074/jbc.M113.539148 24719317 PMC4140899

[B21] Cevoli S. Mochi M. Scapoli C. Marzocchi N. Pierangeli G. Pini L.. (2006). A genetic association study of dopamine metabolism-related genes and chronic headache with drug abuse. Eur. J. Neurol. 13, 1009–1013. 10.1111/j.1468-1331.2006.01415.x 16930369

[B22] Chaplan S. R. Bach F. W. Pogrel J. W. Chung J. M. Yaksh T. L. (1994). Quantitative assessment of tactile allodynia in the rat paw. J. Neurosci. Methods 53, 55–63. 10.1016/0165-0270(94)90144-9 7990513

[B23] Christiansen I. Thomsen L. L. Daugaard D. Ulrich V. Olesen J. (1999). Glyceryl trinitrate induces attacks of migraine without aura in sufferers of migraine with aura. Cephalalgia 19, 660–667; discussion 626. 10.1046/j.1468-2982.1999.019007660.x 10524660

[B24] Cury Y. Picolo G. Gutierrez V. P. Ferreira S. H. (2011). Pain and analgesia: the dual effect of nitric oxide in the nociceptive system. Nitric Oxide 25, 243–254. 10.1016/j.niox.2011.06.004 21723953

[B25] D'Andrea G. Leon A. (2010). Pathogenesis of migraine: from neurotransmitters to neuromodulators and beyond. Neurol. Sci. 31, S1–S7. 10.1007/s10072-010-0267-8 20464574

[B26] De Felice M. Eyde N. Dodick D. Dussor G. O. Ossipov M. H. Fields H. L.. (2013). Capturing the aversive state of cephalic pain preclinically. Ann. Neurol. 74, 257–265. 10.1002/ana.23922 23686557 PMC3830648

[B27] De Fusco M. Marconi R. Silvestri L. Atorino L. Rampoldi L. Morgante L.. (2003). Haploinsufficiency of ATP1A2 encoding the Na+/K+ pump α2 subunit associated with familial hemiplegic migraine type 2. Nat. Genet. 33, 192–196. 10.1038/ng1081 12539047

[B28] Demartini C. Tassorelli C. Zanaboni A. M. Tonsi G. Francesconi O. Nativi C.. (2017). The role of the transient receptor potential ankyrin type-1 (TRPA1) channel in migraine pain: evaluation in an animal model. J. Headache Pain 18:94. 10.1186/s10194-017-0804-4 28884307 PMC5589714

[B29] Descalzi G. Mitsi V. Purushothaman I. Gaspari S. Avrampou K. Loh Y. E.. (2017). Neuropathic pain promotes adaptive changes in gene expression in brain networks involved in stress and depression. Sci. Signal. 10:eaaj1549. 10.1126/scisignal.aaj1549 28325815 PMC5524975

[B30] Di W. Shi X. Lv H. Liu J. Zhang H. Li Z.. (2016). Activation of the nuclear factor E2-related factor 2/anitioxidant response element alleviates the nitroglycerin-induced hyperalgesia in rats. J. Headache Pain 17:99. 10.1186/s10194-016-0694-x 27778243 PMC5078120

[B31] Dichgans M. Freilinger T. Eckstein G. Babini E. Lorenz-Depiereux B. Biskup S.. (2005). Mutation in the neuronal voltage-gated sodium channel SCN1A in familial hemiplegic migraine. Lancet 366, 371–377. 10.1016/S0140-6736(05)66786-4 16054936

[B32] D'Souza C. A. Chopra V. Varhol R. Xie Y. Y. Bohacec S. Zhao Y.. (2008). Identification of a set of genes showing regionally enriched expression in the mouse brain. BMC Neurosci. 9:66. 10.1186/1471-2202-9-66 18625066 PMC2483290

[B33] Du X. Hao H. Yang Y. Huang S. Wang C. Gigout S.. (2017). Local GABAergic signaling within sensory ganglia controls peripheral nociceptive transmission. J. Clin. Investigation 127, 1741–1756. 10.1172/JCI86812 28375159 PMC5409786

[B34] Eising E. Huisman S. M. Mahfouz A. Vijfhuizen L. S. Anttila V. Winsvold B. S.. (2016). Gene co-expression analysis identifies brain regions and cell types involved in migraine pathophysiology: a GWAS-based study using the Allen Human Brain Atlas. Hum. Genet. 135, 425–439. 10.1007/s00439-016-1638-x 26899160 PMC4796339

[B35] Eising E. Shyti R. AC't Hoen P. Vijfhuizen L. S. Huisman S. M. Broos L. A.. (2017). Cortical spreading depression causes unique dysregulation of inflammatory pathways in a transgenic mouse model of migraine. Mol. Neurobiol. 54, 2986–2996. 10.1007/s12035-015-9681-5 27032388 PMC5390001

[B36] Elman I. Borsook D. (2016). Common brain mechanisms of chronic pain and addiction. Neuron 89, 11–36. 10.1016/j.neuron.2015.11.027 26748087

[B37] Farajdokht F. Babri S. Karimi P. Alipour M. R. Bughchechi R. Mohaddes G. (2017). Chronic ghrelin treatment reduced photophobia and anxiety-like behaviors in nitroglycerin- induced migraine: role of pituitary adenylate cyclase-activating polypeptide. Eur. J. Neurosci. 45, 763–772. 10.1111/ejn.13486 27886414

[B38] Ferrari L. F. Levine J. D. Green P. G. (2016). Mechanisms mediating nitroglycerin-induced delayed-onset hyperalgesia in the rat. Neuroscience 317, 121–129. 10.1016/j.neuroscience.2016.01.005 26779834 PMC4738056

[B39] Ferrari M. D. Klever R. R. Terwindt G. M. Ayata C. van den Maagdenberg A. M. (2015). Migraine pathophysiology: lessons from mouse models and human genetics. Lancet Neurol. 14, 65–80. 10.1016/S1474-4422(14)70220-0 25496898

[B40] García-Martín E. Martínez C. Serrador M. Alonso-Navarro H. Navacerrada F. Agúndez J. A.. (2014). SLC1A2 rs3794087 variant and risk for migraine. J. Neurol. Sci. 338, 92–95. 10.1016/j.jns.2013.12.022 24412224

[B41] Gasparini C. F. Smith R. A. Griffiths L. R. (2016). Genetic insights into migraine and glutamate: a protagonist driving the headache. J. Neurol. Sci. 367, 258–268. 10.1016/j.jns.2016.06.016 27423601

[B42] Gonzalez-Pena D. Nixon S. E. O'Connor J. C. Southey B. R. Lawson M. A. McCusker R. H.. (2016a). Microglia transcriptome changes in a model of depressive behavior after immune challenge. PLoS ONE 11:e0150858. 10.1371/journal.pone.0150858 26959683 PMC4784788

[B43] Gonzalez-Pena D. Nixon S. E. Southey B. R. Lawson M. A. McCusker R. H. Hernandez A. G.. (2016b). Differential transcriptome networks between IDO1-knockout and wild-type mice in brain microglia and macrophages. PLoS ONE 11:e0157727. 10.1371/journal.pone.0157727 27314674 PMC4912085

[B44] Gormley P. Winsvold B. S. Nyholt D. R. Kallela M. Chasman D. I. Palotie A. (2016). Migraine genetics: from genome-wide association studies to translational insights. Genome Med. 8:86. 10.1186/s13073-016-0346-4 27543003 PMC4992240

[B45] Granella F. Farina S. Malferrari G. Manzoni G. C. (1987). Drug abuse in chronic headache: a clinico-epidemiologic study. Cephalalgia 7, 15–19. 10.1046/j.1468-2982.1987.0701015.x 3581158

[B46] Greco R. Meazza C. Mangione A. S. Allena M. Bolla M. Amantea D.. (2011). Temporal profile of vascular changes induced by systemic nitroglycerin in the meningeal and cortical districts. Cephalalgia 31, 190–198. 10.1177/0333102410379887 20693231

[B47] Greco R. Tassorelli C. Cappelletti D. Sandrini G. Nappi G. (2005). Activation of the transcription Factor NF-κB in the nucleus trigeminalis caudalis in an animal model of migraine. Neurotoxicology 26, 795–800. 10.1016/j.neuro.2005.02.005 15936821

[B48] Hering R. Kuritzky A. (1992). Sodium valproate in the prophylactic treatment of migraine: a double-blind study versus placebo. Cephalalgia 12, 81–84. 10.1046/j.1468-2982.1992.1202081.x 1576648

[B49] Huang D. W. Sherman B. T. Lempicki R. A. (2009). Systematic and integrative analysis of large gene lists using DAVID bioinformatics resources. Nature Protocols 4:44 10.1038/nprot.2008.211 19131956

[B50] Irie O. Kosaka T. Ehara T. Yokokawa F. Kanazawa T. Hirao H.. (2008). Discovery of orally bioavailable cathepsin S inhibitors for the reversal of neuropathic pain. J. Med. Chem. 51, 5502–5505. 10.1021/jm800839j 18754655

[B51] Iversen H. K. Olesen J. Tfelt-Hansen P. (1989). Intravenous nitroglycerin as an experimental model of vascular headache. Basic characteristics. Pain 38, 17–24. 10.1016/0304-3959(89)90067-5 2506503

[B52] Jiang B.-C. He L.-N. Wu X.-B. Shi H. Zhang W.-W. Zhang Z.-J.. (2017). Promoted interaction of C/EBPα with demethylated Cxcr3 gene promoter contributes to neuropathic pain in mice. J. Neurosci. 37, 685–700. 10.1523/JNEUROSCI.2262-16.2016 28100749 PMC6596757

[B53] Johannessen C. U. Johannessen S. I. (2003). Valproate: past, present, and future. CNS Drug Rev. 9, 199–216. 10.1111/j.1527-3458.2003.tb00249.x 12847559 PMC6741662

[B54] Karatas H. Erdener S. E. Gursoy-Ozdemir Y. Lule S. Eren-Kocak E. Sen Z. D.. (2013). Spreading depression triggers headache by activating neuronal Panx1 channels. Science 339, 1092–1095. 10.1126/science.1231897 23449592

[B55] Langemark M. Bach F. Ekman R. Olesen J. (1995). Increased cerebrospinal fluid met-enkephalin immunoreactivity in patients with chronic tension-type headache. Pain 63, 103–107. 10.1016/0304-3959(95)00020-S 8577479

[B56] LaPaglia D. M. Sapio M. R. Burbelo P. D. Thierry-Mieg J. Thierry-Mieg D. Raithel S. J.. (2018). RNA-Seq investigations of human post-mortem trigeminal ganglia. Cephalalgia. 38, 912–932. 28699403 10.1177/0333102417720216 PMC6326384

[B57] Long T. He W. Pan Q. Zhang S. Zhang Y. Liu C.. (2018). Microglia P2X4 receptor contributes to central sensitization following recurrent nitroglycerin stimulation. J. Neuroinflamm. 15:245. 10.1186/s12974-018-1285-3 30165876 PMC6117935

[B58] Maneepak M. Le Grand S. Srikiatkhachorn A. (2009). Serotonin depletion increases nociception-evoked trigeminal NMDA receptor phosphorylation. Headache J. Head Face Pain 49, 375–382. 10.1111/j.1526-4610.2009.01341.x 19220502

[B59] Maratou K. Wallace V. C. Hasnie F. S. Okuse K. Hosseini R. Jina N.. (2009). Comparison of dorsal root ganglion gene expression in rat models of traumatic and HIV-associated neuropathic pain. Eur. J. Pain 13, 387–398. 10.1016/j.ejpain.2008.05.011 18606552 PMC2706986

[B60] Marchenkova A. van den Maagdenberg A. M. Nistri A. (2016a). Loss of inhibition by brain natriuretic peptide over P2X3 receptors contributes to enhanced spike firing of trigeminal ganglion neurons in a mouse model of familial hemiplegic migraine type-1. Neuroscience 331, 197–205. 10.1016/j.neuroscience.2016.06.034 27346147

[B61] Marchenkova A. Vilotti S. Ntamati N. van den Maagdenberg A. M. Nistri A. (2016b). Inefficient constitutive inhibition of P2X3 receptors by brain natriuretic peptide system contributes to sensitization of trigeminal sensory neurons in a genetic mouse model of familial hemiplegic migraine. Mol. Pain 12. 10.1177/1744806916646110 27175010 PMC4955999

[B62] Markovics A. Kormos V. Gaszner B. Lashgarara A. Szoke E. Sandor K.. (2012). Pituitary adenylate cyclase-activating polypeptide plays a key role in nitroglycerol-induced trigeminovascular activation in mice. Neurobiol. Dis. 45, 633–644. 10.1016/j.nbd.2011.10.010 22033344

[B63] McBride W. J. Schultz J. A. Kimpel M. W. McClintick J. N. Wang M. You J.. (2009). Differential effects of ethanol in the nucleus accumbens shell of alcohol-preferring (P), alcohol-non-preferring (NP) and Wistar rats: a proteomics study. Pharmacol. Biochem. Behav. 92, 304–313. 10.1016/j.pbb.2008.12.019 19166871 PMC2643313

[B64] Mongin A. A. (2016). Volume-regulated anion channel–a frenemy within the brain. Pflugers Arch. 468, 421–441. 10.1007/s00424-015-1765-6 26620797 PMC4752865

[B65] Moye L. S. Pradhan A. A. A. (2017). Animal model of chronic migraine-associated pain. Curr. Protoc. Neurosci. 80, 9.60.61–69.60.69. 10.1002/cpns.33 28678396 PMC5558838

[B66] Mulleners W. M. Chronicle E. P. (2008). Anticonvulsants in migraine prophylaxis: a Cochrane review. Cephalalgia 28, 585–597. 10.1111/j.1468-2982.2008.01571.x 18454787

[B67] Nixon S. E. Gonzalez-Pena D. Lawson M. A. McCusker R. H. Hernandez A. G. O'Connor J. C.. (2015). Analytical workflow profiling gene expression in murine macrophages. J. Bioinform. Comput. Biol. 13:1550010. 10.1142/S0219720015500109 25708305 PMC4539142

[B68] Ofte H. K. Berg D. H. Bekkelund S. I. Alstadhaug K. B. (2013). Insomnia and periodicity of headache in an arctic cluster headache population. Headache J Head Face Pain 53, 1602–1612. 10.1111/head.12241 24266336

[B69] Olesen J. (2008). The role of nitric oxide (NO) in migraine, tension-type headache and cluster headache. Pharmacol. Ther. 120, 157–171. 10.1016/j.pharmthera.2008.08.003 18789357

[B70] Olesen J. (2010). Nitric oxide-related drug targets in headache. Neurotherapeutics 7, 183–190. 10.1016/j.nurt.2010.03.006 20430317 PMC5084099

[B71] Ophoff R. A. Terwindt G. M. Vergouwe M. N. Van Eijk R. Oefner P. J. Hoffman S. M. (1996). Familial hemiplegic migraine and episodic ataxia type-2 are caused by mutations in the Ca 2+ channel gene CACNL1A4. Cell 87, 543–552. 10.1016/S0092-8674(00)81373-2 8898206

[B72] Packard R. C. Ham L. P. (1997). Pathogenesis of posttraumatic headache and migraine: a common headache pathway? Headache J. Head Face Pain 37, 142–152. 10.1046/j.1526-4610.1997.3703142.x 9100398

[B73] Pattyn A. Simplicio N. van Doorninck J. H. Goridis C. Guillemot F. Brunet J.-F. (2004). Ascl1/Mash1 is required for the development of central serotonergic neurons. Nat. Neurosci. 7, 589–595. 10.1038/nn1247 15133515

[B74] Pedersen S. H. Maretty L. Ramachandran R. Sibbesen J. A. Yakimov V. Elgaard-Christensen R. (2016). RNA sequencing of trigeminal ganglia in rattus norvegicus after glyceryl trinitrate infusion with relevance to migraine. PLoS ONE 11:e0155039 10.1371/journal.pone.0155039 27213950 PMC4877077

[B75] Pietrobon D. (2013). Calcium channels and migraine. Biochim. Biophys. Acta Biomembr. 1828, 1655–1665. 10.1016/j.bbamem.2012.11.012 23165010

[B76] Pradhan A. A. Smith M. L. McGuire B. Tarash I. Evans C. J. Charles A. (2014a). Characterization of a novel model of chronic migraine. PAIN® 155, 269–274. 10.1016/j.pain.2013.10.004 24121068 PMC3920577

[B77] Pradhan A. A. Smith M. L. Zyuzin J. Charles A. (2014b). delta-Opioid receptor agonists inhibit migraine-related hyperalgesia, aversive state and cortical spreading depression in mice. Br. J. Pharmacol. 171, 2375–2384. 10.1111/bph.12591 24467301 PMC3997277

[B78] Pringsheim T. (2002). Cluster headache: evidence for a disorder of circadian rhythm and hypothalamic function. Can. J. Neurol. Sci. 29, 33–40. 10.1017/S0317167100001694 11858532

[B79] Pruitt K. D. Tatusova T. Maglott D. R. (2007). NCBI reference sequences (RefSeq): a curated non-redundant sequence database of genomes, transcripts and proteins. Nucleic Acids Res. 35(Suppl 1), D61–D65. 10.1093/nar/gkl842 17130148 PMC1716718

[B80] Qian A. Song D. Li Y. Liu X. Tang D. Yao W.. (2013). Role of voltage gated Ca2+ channels in rat visceral hypersensitivity change induced by 2, 4, 6-trinitrobenzene sulfonic acid. Molecular Pain 9:15. 10.1186/1744-8069-9-15 23537331 PMC3626538

[B81] Quaegebeur A. Segura I. Schmieder R. Verdegem D. Decimo I. Bifari F.. (2016). Deletion or inhibition of the oxygen sensor PHD1 protects against ischemic stroke via reprogramming of neuronal metabolism. Cell Metab. 23, 280–291. 10.1016/j.cmet.2015.12.007 26774962 PMC4880550

[B82] Reiner A. Yekutieli D. Benjamini Y. (2003). Identifying differentially expressed genes using false discovery rate controlling procedures. Bioinformatics 19, 368–375. 10.1093/bioinformatics/btf877 12584122

[B83] Reuter U. Chiarugi A. Bolay H. Moskowitz M. A. (2002). Nuclear factor-κB as a molecular target for migraine therapy. Ann. Neurol. 51, 507–516. 10.1002/ana.10159 11921057

[B84] Robinson M. D. McCarthy D. J. Smyth G. K. (2010). edgeR: a Bioconductor package for differential expression analysis of digital gene expression data. Bioinformatics 26, 139–140. 10.1093/bioinformatics/btp616 19910308 PMC2796818

[B85] Sarchielli P. Di Filippo M. Nardi K. Calabresi P. (2007). Sensitization, glutamate, and the link between migraine and fibromyalgia. Curr. Pain Headache Rep. 11, 343–351. 10.1007/s11916-007-0216-2 17894924

[B86] Schack V. R. Holm R. Vilsen B. (2012). Inhibition of phosphorylation of Na+, K+-ATPase by mutations causing familial hemiplegic migraine. J. Biol. Chem. 287, 2191–2202. 10.1074/jbc.M111.323022 22117059 PMC3265897

[B87] Schleithoff L. Mehrke G. Reutlinger B. Lehmann-Horn F. (1999). Genomic structure and functional expression of a human alpha(2)/delta calcium channel subunit gene (CACNA2). Genomics 61, 201–209. 10.1006/geno.1999.5941 10534405

[B88] Schwartz N. Temkin P. Jurado S. Lim B. K. Heifets B. D. Polepalli J. S.. (2014). Chronic pain. Decreased motivation during chronic pain requires long-term depression in the nucleus accumbens. Science 345, 535–542. 10.1126/science.1253994 25082697 PMC4219555

[B89] Serão N. V. González-Peña D. Beever J. E. Faulkner D. B. Southey B. R. Rodriguez-Zas S. L. (2013). Single nucleotide polymorphisms and haplotypes associated with feed efficiency in beef cattle. BMC Genetics 14:94. 10.1186/1471-2156-14-94 24066663 PMC3819741

[B90] Shannon P. Markiel A. Ozier O. Baliga N. S. Wang J. T. Ramage D.. (2003). Cytoscape: a software environment for integrated models of biomolecular interaction networks. Genome Res. 13, 2498–2504. 10.1101/gr.1239303 14597658 PMC403769

[B91] Silbereis J. C. Nobuta H. Tsai H. H. Heine V. M. McKinsey G. L. Meijer D. H.. (2014). Olig1 function is required to repress dlx1/2 and interneuron production in Mammalian brain. Neuron 81, 574–587. 10.1016/j.neuron.2013.11.024 24507192 PMC3979971

[B92] Solomon G. D. (1991). Circadian rhythms and migraine. Cleveland Clinic J. Med. 59, 326–329. 10.3949/ccjm.59.3.326 1516220

[B93] Soneson C. Love M. I. Robinson M. D. (2015). Differential analyses for RNA-seq: transcript-level estimates improve gene-level inferences. F1000 Res. 4. 10.12688/f1000research.7563.1 26925227 PMC4712774

[B94] Subramanian A. Kuehn H. Gould J. Tamayo P. Mesirov J. P. (2007). GSEA-P: a desktop application for gene set enrichment analysis. Bioinformatics 23, 3251–3253. 10.1093/bioinformatics/btm369 17644558

[B95] Sufka K. J. Staszko S. M. Johnson A. P. Davis M. E. Davis R. E. Smitherman T. A. (2016). Clinically relevant behavioral endpoints in a recurrent nitroglycerin migraine model in rats. J. Headache Pain 17, 1–7. 10.1186/s10194-016-0624-y 27093871 PMC4837195

[B96] Sydow K. Daiber A. Oelze M. Chen Z. August M. Wendt M.. (2004). Central role of mitochondrial aldehyde dehydrogenase and reactive oxygen species in nitroglycerin tolerance and cross-tolerance. J. Clin. Investig. 113, 482–489. 10.1172/JCI200419267 14755345 PMC324536

[B97] Tipton A. F. Tarash I. McGuire B. Charles A. Pradhan A. A. (2016). The effects of acute and preventive migraine therapies in a mouse model of chronic migraine. Cephalalgia 36, 1048–1056. 10.1177/0333102415623070 26682574 PMC5557701

[B98] Torta D. M. Costa T. Luda E. Barisone M. G. Palmisano P. Duca S.. (2016). Nucleus accumbens functional connectivity discriminates medication-overuse headache. Neuroimage Clin. 11, 686–693. 10.1016/j.nicl.2016.05.007 27330969 PMC4900511

[B99] Vadasz C. Saito M. O'Brien D. Zavadil J. Morahan G. Chakraborty G.. (2007). Ventral tegmental transcriptome response to intermittent nicotine treatment and withdrawal in BALB/cJ, C57BL/6ByJ, and quasi-congenic RQI mice. Neurochem. Res. 32, 457–480. 10.1007/s11064-006-9250-4 17268848

[B100] van Dongen R. M. Zielman R. Noga M. Dekkers O. M. Hankemeier T. van den Maagdenberg A. M.. (2017). Migraine biomarkers in cerebrospinal fluid: a systematic review and meta-analysis. Cephalalgia 37, 49–63. 10.1177/0333102415625614 26888294

[B101] Venkatakrishnan A. Deupi X. Lebon G. Tate C. G. Schertler G. F. Babu M. M. (2013). Molecular signatures of G-protein-coupled receptors. Nature 494, 185–194. 10.1038/nature11896 23407534

[B102] Verfaillie A. Imrichová H. Van de Sande B. Standaert L. Christiaens V. Hulselmans G.. (2014). iRegulon: from a gene list to a gene regulatory network using large motif and track collections. PLoS Comput. Biol. 10:e1003731. 10.1371/journal.pcbi.1003731 25058159 PMC4109854

[B103] Wheeler H. E. Gamazon E. R. Wing C. Njiaju U. O. Njoku C. Baldwin R. M.. (2013). Integration of cell line and clinical trial genome-wide analyses supports a polygenic architecture of Paclitaxel-induced sensory peripheral neuropathy. Clin. Cancer Res. 19, 491–499. 10.1158/1078-0432.CCR-12-2618 23204130 PMC3549006

[B104] Yau H.-J. Wang H.-F. Lai C. Liu F.-C. (2003). Neural development of the neuregulin receptor ErbB4 in the cerebral cortex and the hippocampus: preferential expression by interneurons tangentially migrating from the ganglionic eminences. Cerebral Cortex 13, 252–264. 10.1093/cercor/13.3.252 12571115

[B105] Yuan K. Zhao L. Cheng P. Yu D. Dong T. Xing L.. (2013). Altered structure and resting-state functional connectivity of the basal ganglia in migraine patients without aura. J. Pain 14, 836–844. 10.1016/j.jpain.2013.02.010 23669074

